# Hedgehog signaling can enhance glycolytic ATP production in the *Drosophila* wing disc

**DOI:** 10.15252/embr.202154025

**Published:** 2022-09-22

**Authors:** Ioannis Nellas, K Venkatesan Iyer, Juan M Iglesias‐Artola, Martin Pippel, André Nadler, Suzanne Eaton, Natalie A Dye

**Affiliations:** ^1^ Max Planck Institute for Molecular Cell Biology and Genetics Dresden Germany; ^2^ Excellence Cluster, Physics of Life Technische Universität Dresden Dresden Germany; ^3^ Department of Mechanical Engineering Indian Institute of Science Bangalore India; ^4^ Center for Systems Biology Dresden Dresden Germany; ^5^ Biotechnologisches Zentrum Technische Universität Dresden Dresden Germany; ^6^ Mildred Scheel Nachwuchszentrum (MSNZ) P2, Medical Faculty Technische Universität Dresden Dresden Germany

**Keywords:** adenosine triphosphate, *Drosophila*, glycolysis, Hedgehog, metabolism, Development, Metabolism, Signal Transduction

## Abstract

Adenosine triphosphate (ATP) production and utilization is critically important for animal development. How these processes are regulated in space and time during tissue growth remains largely unclear. We used a FRET‐based sensor to dynamically monitor ATP levels across a growing tissue, using the *Drosophila* larval wing disc. Although steady‐state levels of ATP are spatially uniform across the wing pouch, inhibiting oxidative phosphorylation reveals spatial differences in metabolic behavior, whereby signaling centers at compartment boundaries produce more ATP from glycolysis than the rest of the tissue. Genetic perturbations indicate that the conserved Hedgehog signaling pathway can enhance ATP production by glycolysis. Collectively, our work suggests the existence of a homeostatic feedback loop between Hh signaling and glycolysis, advancing our understanding of the connection between conserved developmental patterning genes and ATP production during animal tissue development.

## Graphical abstract

Morphogen signaling in the developing *Drosophila* wing can promote adenosine triphosphate production from glycolysis upon loss of oxidative phosphorylation (OxPhos).

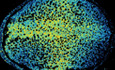

## Introduction

How the production and consumption of energy equivalents is balanced in space and time during animal development remains poorly understood. Tissue growth involves many energy‐consuming processes, including biomolecule synthesis, cytoskeletal remodeling, and intercellular signaling. Such processes can vary considerably in space and time, implying that the production of the energetic currency, adenosine triphosphate (ATP), might also be dynamic. In general, cells can generate ATP via glycolysis, oxidative phosphorylation (OxPhos), or both, depending on context. In the presence of oxygen, OxPhos in mitochondria generates a high yield of ATP. Glycolysis produces less ATP per glucose molecule than OxPhos, but it is anaerobic, cytoplasmic, and fast. Thus, glycolysis can take over during hypoxic conditions or rapidly compensate for short‐timescale fluctuations in energy demand, due to, for example, membrane transport activities (Epstein *et al*, 2014). How these two ATP‐producing processes are regulated in time and space to meet variable energetic demands of a growing tissue is generally not well understood.

In addition to the energetic requirements, animal tissue development also requires morphogens—secreted signals that pattern gene expression across a tissue in a concentration‐dependent manner (Rogers & Schier, 2011). Recently developed methods for monitoring spatial‐temporal patterns in metabolic activity (e.g., Bailey *et al*, 2015; Bulusu *et al*, 2017; Miyazawa *et al*, 2017; Oginuma *et al*, 2017) have enabled studies addressing how morphogen signaling is related to metabolism. In particular, new fluorescent biosensors enable monitoring of metabolite levels at high spatial‐temporal resolution in living cells and tissues (Tsuyama *et al*, 2013; Bulusu *et al*, 2017; Greenwald *et al*, 2018; Volkenhoff *et al*, 2018). Such tools have been used to demonstrate that there is positive feedback between glycolysis and the morphogens FGF/Wnt in the presomitic mesoderm of developing vertebrate embryos (Bulusu *et al*, 2017; Oginuma *et al*, 2017). This feedback is thought to influence cell fate choice during differentiation. Hedgehog (Hh) is another widely conserved morphogen promoting the growth and patterning of different tissues during development and regulating many processes during adult homeostasis (Ingham & McMahon, 2001; Petrova & Joyner, 2014). One of the vertebrate Hh homologs, Sonic Hedgehog (SHH), has been shown to promote glycolysis in fat cells and in cerebellar granule neuron precursors during development and tumorigenesis (Teperino *et al*, 2012; Gershon *et al*, 2013; Di Magno *et al*, 2014). Although these works clearly indicate that morphogen signaling and glycolysis can be coupled with functional consequences for growth and differentiation, it remains unclear whether and how that coupling impacts the overall ATP budget in the tissue.

Here, we use a FRET‐based ATP sensor (Tsuyama *et al*, 2013) to study spatial‐temporal ATP dynamics in the *Drosophila* wing disc, a proliferative tissue that has been a powerful model system for studying principles of morphogen signaling and developmental tissue growth (Hariharan, 2015; Beira & Paro, 2016). The patterns of morphogen signaling are well characterized, easy to visualize in the flat “pouch” region, and able to be genetically perturbed with spatial and temporal control. We exploit these properties to study the interaction between Hh signaling and energy homeostasis in a growing tissue model. We previously showed that steady‐state levels of ATP are uniform across the wing pouch (Spannl *et al*, 2020), even as morphogen signaling and gene expression are exquisitely patterned. Importantly, however, steady‐state measurements may hide differences in rates of ATP production and consumption. For example, it is possible that some regions may consume ATP at a higher rate but also produce ATP at a higher rate, so that the steady‐state levels remain constant across the tissue. Steady‐state measurements also cannot reflect which ATP production pathways, glycolysis versus OxPhos, are involved. To further probe metabolic differences across the tissue, we now dynamically monitor ATP levels after inhibiting ATP production. We find that signaling centers at the compartment boundaries consume ATP slightly faster than elsewhere in the tissue but can also promote ATP production from glycolysis. Genetic perturbations support a role for Hh in positively regulating glycolysis during energy stress. Together with our previous work showing that glycolysis inhibits Hh signaling (Spannl *et al*, 2020), we propose that there is a homeostatic feedback loop between the Hh pathway and glycolysis that buffers fluctuations in ATP levels and morphogen signaling.

## Results and Discussion

### OxPhos inhibition reveals spatially heterogeneous metabolism in the wing disc pouch

**Figure 1:**
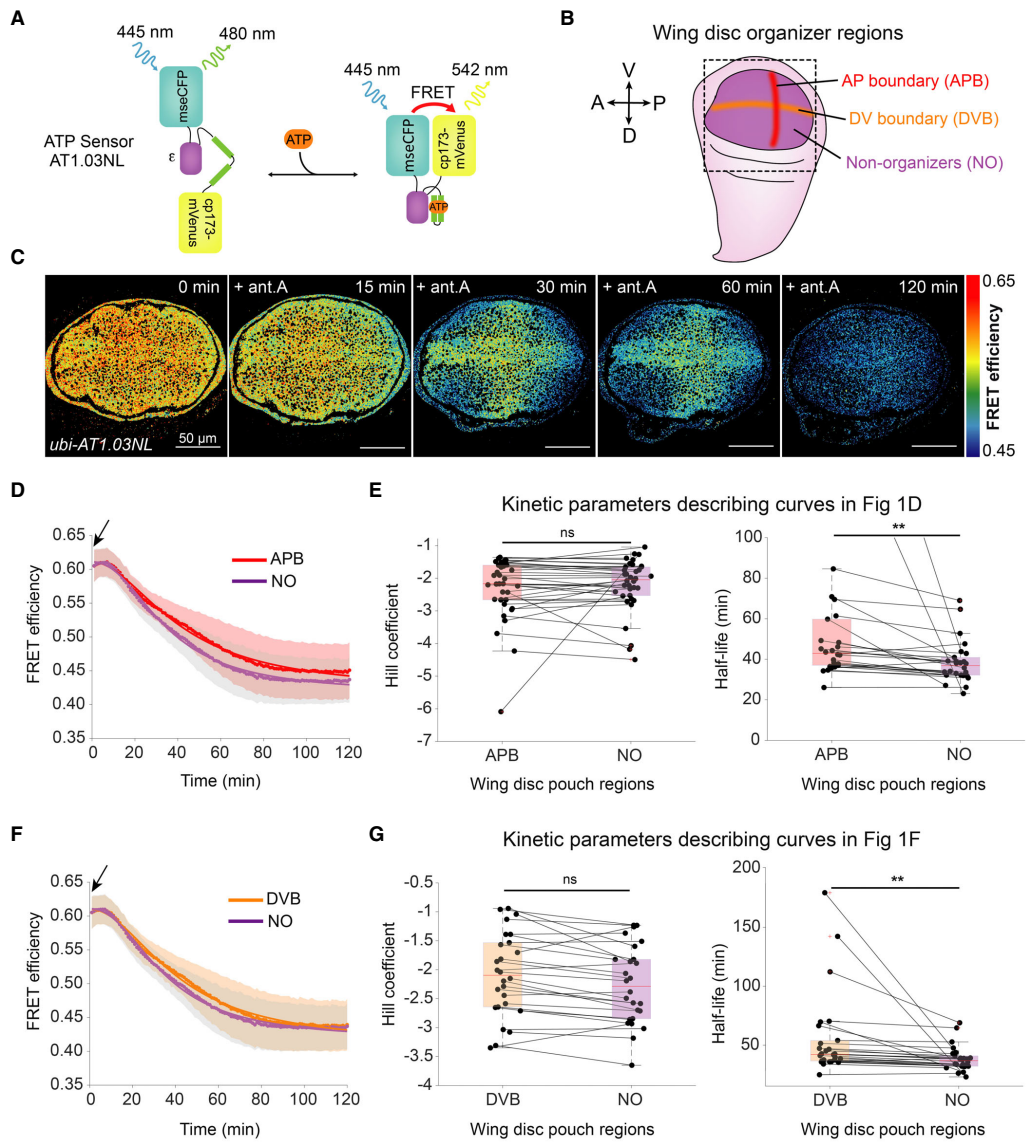
ATP levels decline slower at organizer regions of the wing disc pouch upon OxPhos inhibition A
Schematic of ATP FRET sensor, adapted from (Tsuyama *et al*, 2013). Binding of ATP brings the two fluorophores into close proximity, resulting in FRET.B
Wing disc schematic highlighting organizer and non‐organizer regions in the pouch. V = Ventral, D = Dorsal, A = Anterior, P = Posterior.C
Timelapse of ATP sensor FRET efficiency across the wing disc pouch after addition of 10 μM antimycin A (ant. A).D, F
Mean FRET efficiency measured over time in the (D) AP boundary (APB) or (F) DV boundary (DVB) and non‐organizer regions (NO). Shaded regions indicate standard deviation (SD); dots indicate mean per timepoint, and solid lines indicate the fit to the mean. Black arrows indicate the addition of the drug.E, G
Fit parameters of individual time traces for AP boundary and non‐organizer regions (E) or DV boundary and non‐organizer regions (G). Each dot represents data from one disc, and black lines connect the corresponding regions of the same disc. Box plots summarize the data: boxes encompass the 2^nd^–3^rd^ quartiles, with whiskers indicating the 1^st^ and 4^th^ quartiles and the red line indicating the median. ns = not significant *P*‐value **P‐value < 0.01 using a Kruskal–Wallis test (*n* = 26 discs).

**Figure EV1:**
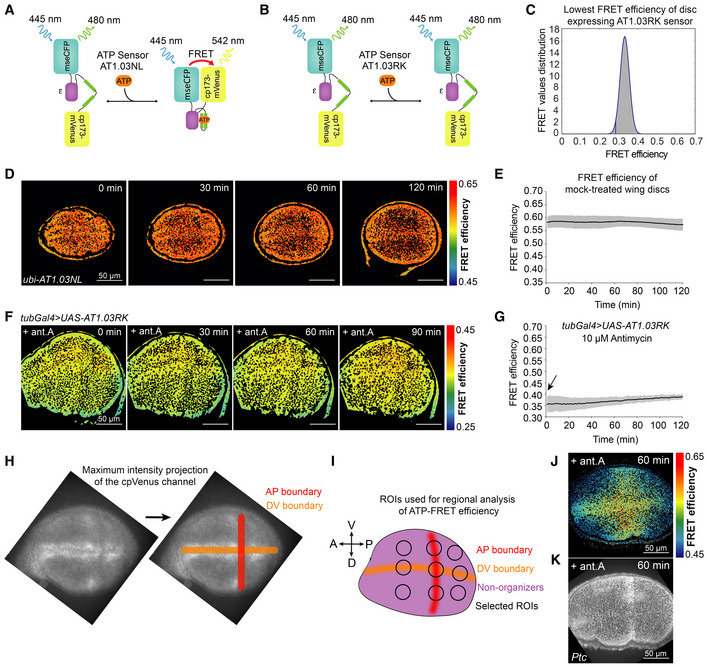
ATP levels in the wing disc pouch are spatially uniform and stable in culture without OxPhos inhibition A, B
Schematic of ATP‐FRET sensor (AT1.03NL, A) and its ATP‐insensitive version (AT1.03RK, B).C
Histogram showing the distribution of FRET efficiency values across a 70‐plane z‐stack of a morphologically healthy, unperturbed wing disc ubiquitously expressing the AT1.03RK sensor: gray area corresponds to the FRET values, and the blue curved line shows a Gaussian fit. The lowest FRET efficiency value used for data fitting was 0.2827 (blue vertical line), defined as the mean FRET efficiency minus twice the standard deviation (SD).D
Timelapse montage of ATP‐FRET sensor efficiency in the wing disc pouch during culture for 2 h without any drugs.E
Mean FRET efficiency in the entire pouch measured over time; gray shade indicates SD, and small black dots represent the means per timepoint (*n* = 9).F
Timelapse montage of FRET efficiency in the wing disc pouch expressing the ATP‐insensitive construct (AT1.03RK) upon addition of 10 μM antimycin A (ant.A).G
Mean FRET efficiency in the entire pouch in (F). Black arrow indicates the addition of the drug; gray shade indicates SD, and small black dots represent the means per timepoint (*n* = 9).H
Anterior‐posterior (AP) and Dorsal‐ventral (DV) boundary regions are discernible with a maximum intensity projection of the cpVenus channel. The AP boundary appears as a stripe of slightly lower intensity, and the DV boundary lies in the middle of two stripes of higher intensity.I
Schematic indicating the location of the ROIs that were used to calculate mean FRET efficiency in different wing pouch regions. The three ROIs in the AP boundary or in the DV boundary were averaged together to calculate the mean FRET efficiency of the AP boundary and DV boundary, respectively. The organizer region was measured as the average of all five of these ROIs (AP + DV boundaries). The non‐organizer region corresponds to the remaining four ROIs outside of the AP + DV boundaries. To verify that the region of slower kinetics of ATP loss upon antimycin addition corresponds to the Hh signaling domain, we fixed discs in the middle of antimycin treatment (after 60 min) and stained for Patched (Ptc).J
Spatial pattern of FRET efficiency after 60 min of 10 μM antimycin A (ant.A) exposure.K
Ptc expression in the same disc as (J) after fixation and immunofluorescence.

We monitored ATP levels across the wing disc pouch in live explants using a ubiquitously expressed FRET‐based ATP sensor, ubi‐AT1.03NL (Fig 1A and B; Tsuyama *et al*, 2013). As previously shown (Spannl *et al*, 2020), steady‐state levels of ATP are similar throughout the wing pouch and decline considerably upon OxPhos inhibition with antimycin A, indicating that OxPhos is a major producer of ATP in the wing disc (Fig 1C). After 2 h of treatment with antimycin A, ATP levels fall close to the lower detection limit of the sensor (as determined by the use of the ATP‐insensitive sensor ubi‐AT1.03RK, Fig EV1A–C, F and G), whereas ATP levels in control wing discs remain stable over this time period (Fig EV1D and E). Interestingly, a transient pattern emerges in the wing pouch during OxPhos inhibition (Fig 1C). ATP levels drop slightly slower in two perpendicular stripe regions, corresponding to the dorsal‐ventral (DV) boundary and an area just anterior to the anterior–posterior (AP) boundary (Fig 1B), as marked by Patched (Ptc) when fixed and stained during OxPhos inhibition (Fig EV1H, J and K). These areas are known growth “organizer” regions, with high signaling activity in the Wingless/Notch and Hedgehog pathways, respectively (Tabata & Takei, 2004; Beira & Paro, 2016). This pattern of ATP decline upon OxPhos inhibition does not occur with the ATP‐insensitive sensor (Fig EV1F and G).

To quantitatively compare the kinetics of ATP depletion in different regions, we locally measured FRET efficiency over time during OxPhos inhibition (Figs 1D and F, and EV1H and I) and fit the data to a four‐parametric logistic curve (see Materials and Methods). To compare the kinetics of ATP depletion in different tissue regions, we compare relative values of two parameters: the time needed to reach half of the FRET efficiency (half‐life, units of time) and the slope of the curve at the half‐life (Hill coefficient, unitless). We find larger half‐life values for the AP and DV boundary regions than for the rest of the pouch (Fig 1E and G), indicating that the AP and DV organizer regions have significantly slower kinetics of ATP depletion upon OxPhos inhibition than the rest of the wing pouch.

### Organizer regions can enhance glycolytic ATP production

Our data from Fig 1 indicate that although steady‐state levels of ATP are spatially uniform, differences in metabolism between organizers and non‐organizers are revealed when ATP production from OxPhos is blocked. If OxPhos is the only active ATP production pathway, we would assume that differences in the rates of ATP decline in these regions reflect differences in consumption rates. It is known, however, that glycolytic genes are expressed during normal wing disc development and have phenotypes with respect to ATP levels, Hh signaling, and tissue growth (Dye *et al*, 2017; Spannl *et al*, 2020). Thus, an alternative explanation is that the organizer regions are better able to compensate from the loss of OxPhos by using glycolysis. To determine whether ATP declines slower in organizer regions upon OxPhos inhibition because they have slower consumption rates or because they do more glycolysis, we examined the kinetics of ATP decline after simultaneous inhibition of glycolysis and OxPhos. If organizer regions produce more ATP from glycolysis, then ATP levels should drop more uniformly across the disc. Any spatial variations in the rate of ATP decline when both production pathways are inhibited can be attributed to variations in the rate of ATP consumption.

**Figure 2:**
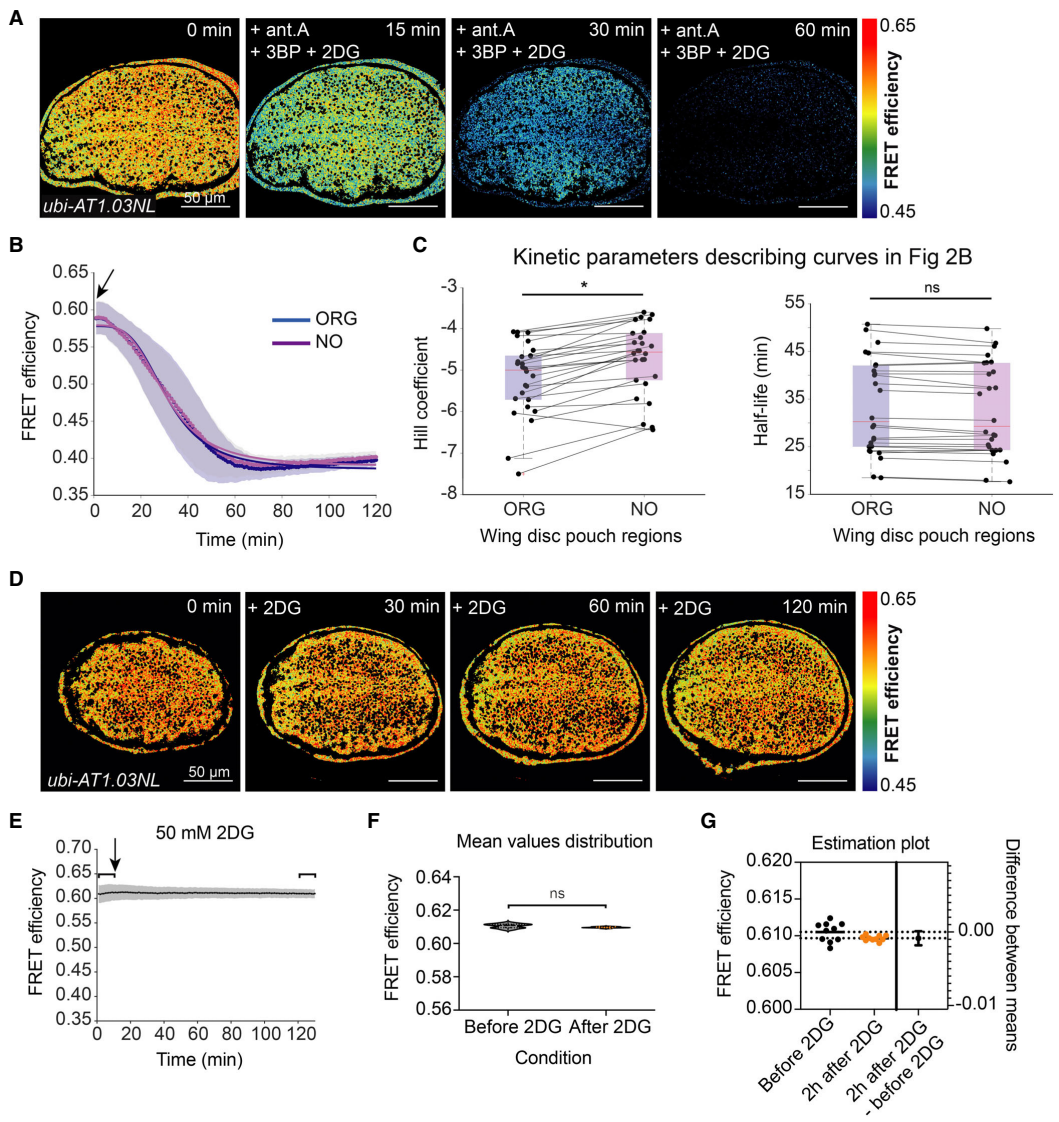
Glycolysis is required for spatial differences in kinetics of ATP decline upon OxPhos inhibition A
Timelapse of ATP sensor FRET efficiency after combined addition of 10 μM antimycin A (ant.A), 100 μM 3‐Bromopyruvate (3BP), and 50 mM 2‐deoxy‐D‐glucose (2DG).B
Mean FRET efficiency of organizer (ORG) regions and non‐organizer (NO) regions analyzed over time. Shaded regions indicate standard deviation (SD); dots indicate mean per timepoint, and solid lines indicate the fit to the mean.C
Fit parameters of individual time traces for organizer and non‐organizer regions. Each dot represents data from one disc, and lines connect the corresponding regions of the same disc. Box plots summarize the data: boxes encompass the 2^nd^–3^rd^ quartiles, with whiskers indicating the 1^st^ and 4^th^ quartiles and the red line indicating the median. *P‐value < 0.05, ns = not significant *P*‐value using a Kruskal–Wallis test (*n* = 26 discs).D
Time lapse montage of ATP sensor FRET efficiency in the wing pouch after 2DG addition.E–G
Mean FRET efficiency time trace of the entire wing pouch; shaded region indicates SD; black arrows indicate the addition of the drugs. Brackets indicate the mean FRET values before and 2 h after 2DG addition (1–10 min and 121–130 min, respectively). The distribution of mean values within these brackets are shown as violin plots in (F) (solid line indicates median, dotted indicate quartiles). These were compared using an unpaired *t*‐test (ns = not significant *P*‐value, *n* = 17 discs) including (G) Welch's correction and estimation plot (*n* = 17 discs for each group). In (G), horizontal dotted lines run through the mean values for the two samples (before 2DG and 2 h after 2DG); on the right, the difference between the two means is plotted on a separate axis (right), where the bars indicate the upper and lower 95% confidence intervals.

As intermediate metabolites can enter glycolysis at different steps, we inhibited glycolysis with a combination of two drugs: 3‐bromo‐pyruvate (3BP), which inhibits glycolysis at the step of Glyceraldehyde 3‐phosphate dehydrogenase (Gapdh), and 2‐deoxy‐D‐glucose (2DG), which acts at the level of Hexokinase and Phosphoglucose isomerase. We found that the addition of glycolytic inhibitors with the OxPhos inhibitor antimycin A significantly weakens the pattern observed with antimycin A alone (Fig 2A): ATP levels decay in organizer and non‐organizer regions with similar kinetics (Fig 2B and C). Half‐life values are indistinguishable, but interestingly, the Hill coefficient is slightly lower in the organizer regions than elsewhere in the tissue (Fig 2C). The latter indicates a slightly faster depletion of ATP in organizers, suggesting that these regions actually consume ATP at a slightly faster rate than elsewhere. Thus, the slower decline of ATP in organizer regions upon OxPhos inhibition (Fig 1) cannot be attributed to a slower consumption rate, and we conclude that organizer regions produce more ATP from glycolysis during OxPhos inhibition than the rest of the wing disc pouch.

**Figure EV2:**
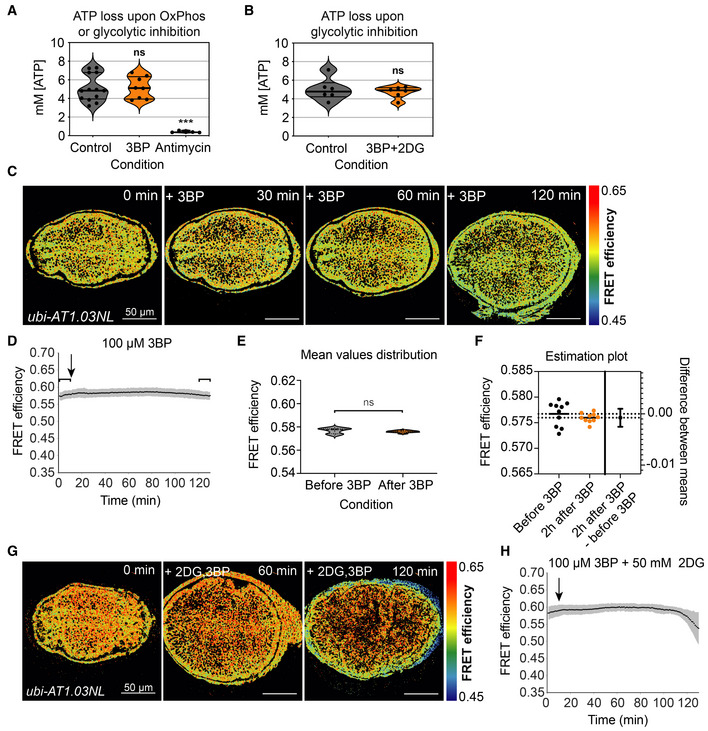
Glycolysis inhibitors alone do not significantly affect ATP levels A
ATP levels measured using a luminescence‐based biochemical assay from single discs after a 2 h treatment with either 50 μM 3‐bromopyruvate (3BP) (*n* = 8) or 10 μM antimycin A (*n* = 5) compared to untreated control discs (*n* = 13). ***P‐value < 0.001, ns = not significant using either Mann–Whitney test (control vs. 3BP) or unpaired *t*‐test (control vs. antimycin A).B
ATP levels of single discs after 1 h of treatment with 50 μM 3BP + 50 mM 2‐deoxy‐D‐glucose (2DG) (*n* = 6) compared to untreated control discs (*n* = 6). ns = not significant *P*‐value using a paired *t*‐test. In (A) and (B), the frequency distributions of the data are shown in violin plots, with the thick black horizontal line indicating the median and the thinner lines indicating the quartiles.C
Timelapse montage of ATP‐FRET sensor efficiency after 3BP addition.D–F
Mean FRET efficiency time trace of the entire wing pouch; shaded region indicates standard deviation (SD); black arrows indicate the addition of the drug. Brackets indicate the mean FRET values before and 2 h after 3BP addition (1–10 and 121–130 min, respectively). The distribution of mean values within these brackets are shown as violin plots in (E) (solid line indicates median, dotted indicate quartiles). These were compared using an unpaired *t*‐test (ns = not significant *P*‐value, *n* = 11 discs) including (F) Welch's correction and estimation plot (*n* = 11 discs for each group). In (F), horizontal dotted lines run through the mean values for the two samples (before 3BP and 2 h after 3BP); on the right, the difference between the two means is plotted on a separate axis (right), where the bars indicate the upper and lower 95% confidence intervals.G
Timelapse montage of ATP‐FRET sensor efficiency after 3BP + 2DG addition.H
Mean FRET efficiency measured over time in the entire wing disc pouch upon addition of 2DG + 3BP. Black line indicates the mean, and gray shade indicates SD (*n* = 9). Black arrows indicate the addition of the drugs.

Note that the kinetics of ATP depletion throughout the wing pouch are faster upon inhibition of both OxPhos and glycolysis than upon inhibition of OxPhos alone, as indicated by a comparison of the Hill coefficients in Figs 1 and 2: −4.57 in non‐organizers and −5 in organizers in all drugs (Fig 2C), compared to −2.3 in non‐organizers and around −2.1 in both AP and DV boundaries in antimycin alone (Fig 1E and G). This finding indicates that upon OxPhos inhibition, glycolysis still generates ATP in the entire wing pouch but more so in the organizer regions. We were unable, however, to detect any difference in ATP levels with the FRET reporter upon exposure to glycolytic inhibitors 3BP and 2DG alone or in combination, except at long times at very high concentrations, when tissue integrity starts to look very poor (Figs 2D–G and EV2C–H). Similarly, using a luciferase‐based assay to measure ATP levels in whole wing discs, we did not find any significant effects of glycolytic inhibition (Fig EV2A and B). We interpret these results to mean that most of the ATP in the wing disc is generated by OxPhos and that OxPhos can compensate for the 2 h pharmacological inhibition of glycolysis during *ex vivo* culture. Nonetheless, the depletion of glycolytic enzymes by RNAi over several days *in vivo* results in a reduction of steady‐state ATP levels (Spannl *et al*, 2020). It remains possible that the FRET‐ATP sensor is saturated and therefore cannot detect small drops in ATP upon glycolytic inhibition alone. We do not think this a likely scenario, however, given that most of our experiments start with an ATP FRET efficiency of only ~0.6, but values of up to 0.7 can be observed in the same tissue under similar conditions, suggesting that the sensor can read higher concentrations of ATP.

### Hedgehog signaling enhances ATP production by glycolysis upon OxPhos inhibition

**Figure EV3:**
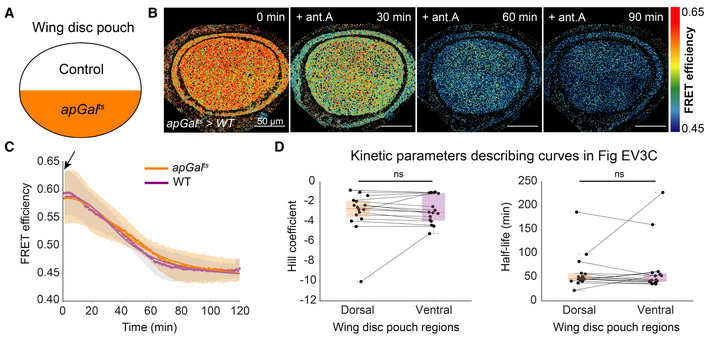
ATP levels decline with similar kinetics in the dorsal and ventral compartments of the *apGal* ^*ts*^ genetic background (without a UAS construct) A
Schematic representation of *apGal* ^*ts*^ expression in the dorsal compartment; ventral compartment serves as an internal control.B
Timelapse montage of ATP sensor FRET efficiency in the wing disc pouch after 10 μM antimycin A (ant.A) addition in *apGal* ^*ts*^ > WT wing discs.C
Mean FRET efficiency measured over time in the dorsal and ventral compartments. Shaded regions indicate standard deviation; dots are the means per timepoint, and solid lines illustrate the fit to the mean.D
Fit parameters of individual time traces for dorsal and ventral compartments shown in (C). Each dot represents data from one disc, and lines connect the corresponding regions of the same disc. Box plots summarize the data: boxes encompass the 2^nd^–3^rd^ quartiles, with whiskers indicating the 1^st^ and 4^th^ quartiles and the red line indicating the median. ns = not significant *P*‐value, using Kruskal–Wallis test (*n* = 16). Black arrow indicates the addition of the drug.

**Figure 3:**
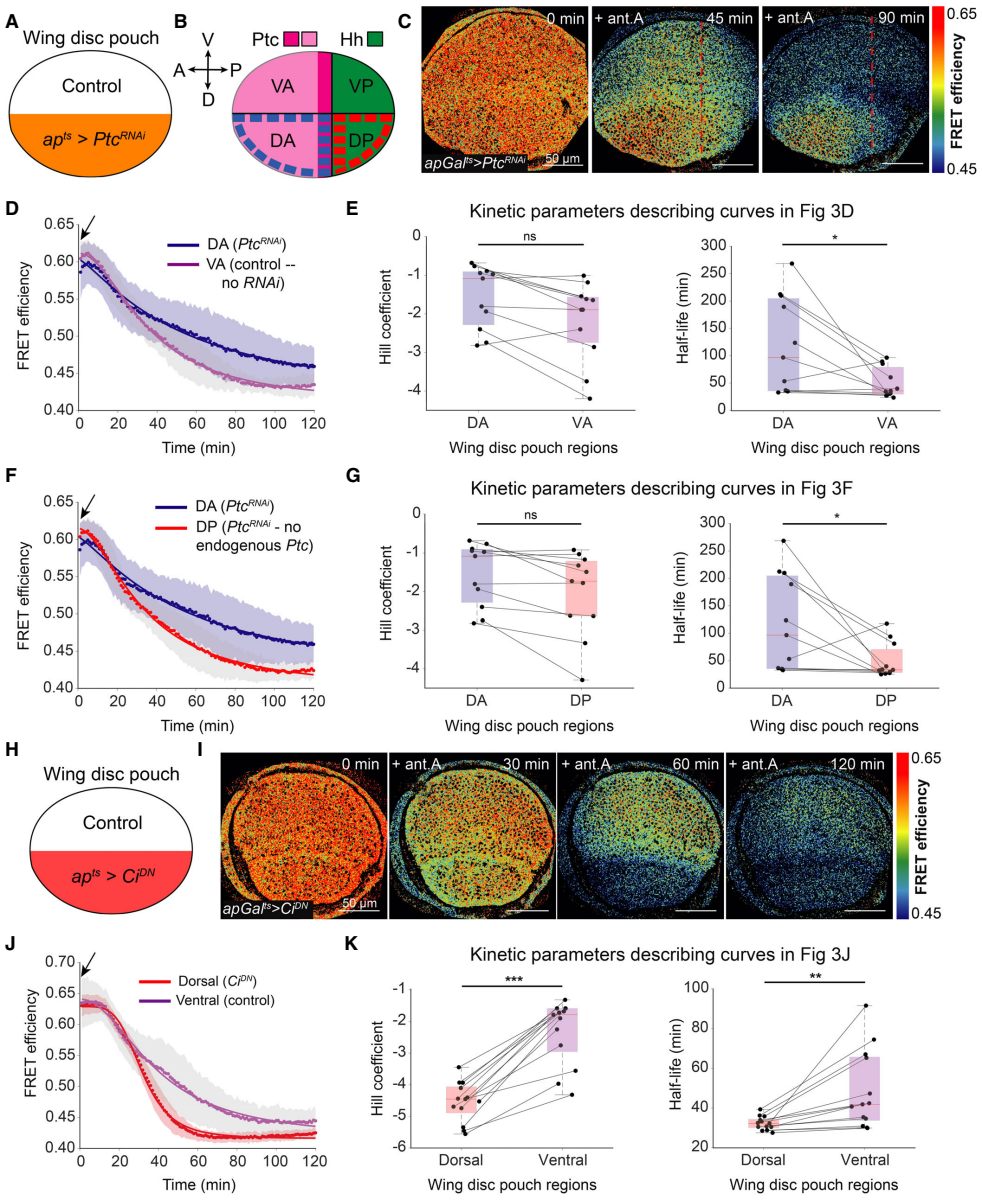
Hedgehog activity promotes glycolytic ATP production upon OxPhos inhibition A, B
Schematics indicating where the RNAi for Ptc was induced using *apGal^ts^* (abbreviated *ap^ts^*) (A) and where Ptc and Hh are normally expressed (B). Dark and light magenta indicate high versus low Ptc expression, respectively. Sub‐compartments enclosed with dashed lines indicate regions analyzed in (D–G). V = Ventral, D = Dorsal, A = Anterior, P = Posterior.C
Timelapse of ATP sensor FRET efficiency in the *apGal* ^*ts*^ > *Ptc* ^*RNAi*^ wing disc pouch after 10 μM antimycin A (ant.A) addition. Red dashed line indicates the position of the AP boundary.D, F
Mean FRET efficiency analyzed over time in the (D) dorsal anterior (DA) and ventral anterior (VA) sub‐compartments or the (F) dorsal anterior (DA) and dorsal posterior (DP) sub‐compartments of *apGal* ^*ts*^ > *Ptc* ^*RNAi*^ wing discs. Shaded regions indicate standard deviation (SD); dots represent the mean per timepoint; solid lines illustrate a fit of the mean data.E, G
Kinetic parameters of the fit line for ATP decline in the DA and VA compartments (E) or DA and DP compartments (G). Lines connect the corresponding regions of the same disc; dots represent the average per timepoint; box plots summarize the data: boxes encompass the 2^nd^–3^rd^ quartiles, with whiskers indicating the 1^st^ and 4^th^ quartiles and the red line indicating the median. *P‐value < 0.05, ns = not significant *P*‐value using a Kruskal–Wallis test (*n* = 11 discs).H
Schematic showing the dorsal expression of the dominant negative Ci allele (Ci^DN^) using *apGal* ^*ts*^.I
Timelapse of ATP sensor FRET efficiency in *apGal* ^*ts*^ > *Ci* ^*DN*^ wing discs after 10 μM antimycin A addition.J
Mean FRET efficiency measured over time in the dorsal and ventral compartments in *apGal* ^*ts*^ > *Ci* ^*DN*^ wings. Shaded regions indicate SD; dots represent the average per timepoint; solid lines illustrate a fit of the mean data.K
Kinetic parameters of the fit line for ATP decline in dorsal and ventral compartments. Lines connect the corresponding regions of the same disc; dots represent the average per timepoint; box plots summarize the data: boxes encompass the 2^nd^–3^rd^ quartiles, with whiskers indicating the 1^st^ and 4^th^ quartiles and the red line indicating the median **P‐value < 0.01, ***P‐value < 0.001 using a Kruskal–Wallis test (*n* = 13 discs). Black arrows indicate the addition of the drug.

Our results suggest that the morphogen signaling that defines the organizer regions can influence ATP production in that part of the wing disc. Consistent with our data, it has been previously shown that Notch signaling, which is localized on either side of the DV boundary, can upregulate glycolysis (Slaninova *et al*, 2016). Here, we now examine how ATP production can be affected by the Hh pathway, which organizes growth and patterning along the AP axis. Hh is expressed in the posterior compartment and travels to the anterior compartment, where it is received by the membrane protein Patched (Ptc) (Basler & Struhl, 1994; Tabata & Kornberg, 1994). In the absence of Hh, Ptc represses the 7‐pass signal transducer Smoothened (Denef *et al*, 2000). Hh binding to Ptc relieves this repression, allowing the Gli‐family transcription factor Cubitus interruptus (Ci) to activate target gene expression. Using the *apterous‐Gal4* combined with *tub‐Gal80* ^*ts*^ (*apGal* ^*ts*^), we alter Hh signaling in the dorsal compartment during larval development and assess the effect on energy metabolism, using the ventral compartment as an internal control (Fig 3A and H, see “Note on Gal4 lines” in Materials and Methods; Brand & Perrimon, 1993; del Valle Rodríguez *et al*, 2012). The dorsal and ventral sides of control *apGal* ^*ts*^ wing discs (no UAS construct) exhibit similar ATP kinetics upon OxPhos inhibition (Fig EV3A–D).

**Figure EV4:**
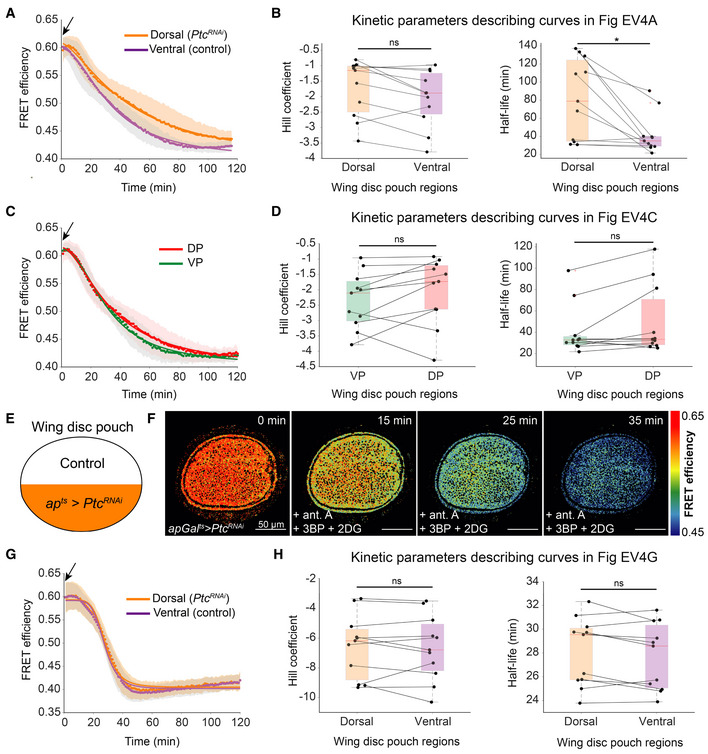
Extended regional analysis of *Ptc* ^*RNAi*^ upon OxPhos inhibition alone or combined with glycolysis inhibition A, C
Mean FRET efficiency measured over time in the dorsal and ventral compartments (A) or the ventral posterior (VP) and dorsal posterior (DP) sub‐compartments (C) of the wing disc pouch in *apGal* ^*ts*^ > *Ptc* ^*RNAi*^ upon OxPhos inhibition. Shaded regions indicate standard deviation (SD); dots are the means per timepoint, and solid lines illustrate a fit to the mean; black arrow indicates the addition of drug.B, D
Fit parameters of individual time traces for the dorsal and ventral compartments (B) or the posterior sub‐compartments (D). Each dot represents data from one disc, and lines connect the corresponding regions of the same disc. Box plots summarize the data: boxes encompass the 2^nd^–3^rd^ quartiles, with whiskers indicating the 1^st^ and 4^th^ quartiles and the red line indicating the median. *P‐value < 0.05, ns = not significant *P‐*value, using a Kruskal–Wallis test (*n* = 11).E
Schematic representation of *apGal* ^*ts*^ > *Ptc* ^*RNAi*^ expression in the dorsal compartment.F
Timelapse montage of ATP‐FRET sensor efficiency after addition of antimycin A (ant.A), 3‐bromopyruvate (3BP), and 2‐deoxy‐D‐glucose (2DG).G
Mean FRET efficiency measured over time in the dorsal and ventral compartments. Shaded regions indicate SD; dots are the means per timepoint, and solid lines illustrate a fit to the mean data; black arrow indicates the addition of drugs.H
Fit parameters of individual time traces for dorsal and ventral compartments. Each dot represents data from one disc, and lines connect the corresponding regions of the same disc. Box plots summarize the data (see Materials and Methods). ns = not significant *P‐*value, using Kruskal–Wallis test (*n* = 12).

**Figure EV5:**
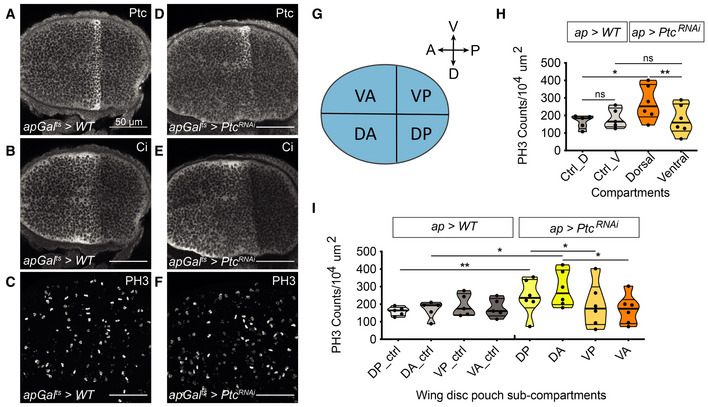
Upregulation of Hh pathway activity with *Ptc* ^*RNAi*^ increases proliferation in both anterior and posterior compartments A–F
Control (*apGal* ^*ts*^ > *WT*, A–C) or *apGal* ^*ts*^ > *Ptc* ^*RNAi*^ (D–F) wing pouch stained for Ptc (A, D), Ci (B, E) or the mitotic marker phospho‐histone H3 (PH3, C, F).G
Schematic of wing disc sub‐compartments. V = Ventral, D = Dorsal, A = Anterior, P = Posterior.H
Quantification of PH3‐positive nuclei in the dorsal (D) and ventral (V) compartments of the pouch of control (Ctrl, *apGal* ^*ts*^ > *WT*, *n* = 5) and *apGal* ^*ts*^ > *Ptc* ^*RNAi*^ (*n* = 6) wing discs. *P‐value < 0.05, **P‐value < 0.01 using, ns = not significant *P*‐value using either paired *t*‐tests between disc compartments belonging to the same group (*apGal* ^*ts*^ > *Ptc* ^*RNAi*^ or control) or Mann–Whitney tests for different groups (Ctrl_D vs. Dorsal, Ctrl_V vs. Ventral). The frequency distributions of the data are shown with violin plots, with the thick black horizontal line indicating the median and the thinner lines indicating the quartiles.I
Quantification of mitotic density in different compartments of the pouch in control (Ctrl, *ap* > *WT*) and *apGal* ^*ts*^ > *Ptc* ^*RNAi*^ wing discs. *P‐value < 0.05, **P‐value < 0.01 using, ns = not significant *P*‐value using one‐way ANOVA tests with Bonferroni *post hoc* correction.

To overactivate the Hh pathway, we used *apGal* ^*ts*^ to induce RNAi against *Ptc*. *Ptc* is normally expressed in the anterior compartment, with a peak near the AP boundary (Figs 3B, EV1K and EV5A). Dorsal downregulation of Ptc does not affect the spatial pattern of steady‐state ATP levels in the wing disc but strikingly alters the pattern of metabolic activity in response to antimycin A (Figs 3C–G and EV4A–D). We observe a significantly longer half‐life value for ATP depletion in the anterior region of the dorsal compartment (DA in Fig 3E, half‐life of 96.7 min) compared to the anterior region of the ventral compartment, where *apGal* ^*ts*^ is not expressed (VA in Fig 3E, half‐life of 33 min). The half‐life for the DA region is also significantly longer than that of the posterior region of the dorsal compartment (DP, half‐life of 33 min, Fig 3G). This difference can be explained by the fact that Ptc is normally only expressed in the anterior compartment, with a peak near the AP boundary (Fig 3B). To confirm that this effect is caused by more ATP being produced by glycolysis, we again used the combination of OxPhos and glycolytic inhibitors. Inhibiting both metabolic pathways eliminated the spatial differences in kinetics of ATP decline (Fig EV4F–H). Thus, OxPhos inhibition reveals that overactivation of Hh signaling can enhance glycolytic ATP production.

Loss of Patched increases the expression of another growth regulator and Hh target, Decapentaplegic (Dpp), which is secreted and migrates bidirectionally through the tissue to promote growth and proliferation on both the anterior and posterior sides (Affolter & Basler, 2007; Restrepo *et al*, 2014). Consistent with an upregulation of Dpp, we found increased proliferation throughout the dorsal compartment in *apGal* ^*ts*^ > *Ptc* ^*RNAi*^ discs, with no statistical difference between the anterior and posterior sides (Fig EV5A–I). It is unlikely, however, that Dpp mediates the effect of Hh overactivation on glycolytic ATP production, as we only see an effect of *Ptc* ^*RNAi*^ in the Hh‐receiving anterior compartment, where Ptc is normally expressed (Figs 3B–G and EV4C and D).

We reduced Hh pathway activity by overexpressing a dominant negative form of the downstream Hh‐responsive transcription factor, Cubitus interruptus (Ci^DN^, also named Ci^Cell^ in Méthot & Basler, 1999; Fig 3H–K). Inducing the expression of Ci^DN^ has the opposite effect of *Ptc* ^*RNAi*^: ATP levels decline faster during OxPhos inhibition than in the control compartment. Both the Hill coefficient and half‐life values are significantly lower in the dorsal compartment than in the ventral (Hill coefficient = −4.5 for dorsal vs. −1.8 for ventral; half‐life = 32 min for dorsal, 42 min for ventral). Thus, downregulation of Hh pathway reduces ATP production from glycolysis. Note that this perturbation, unlike *Ptc* ^*RNAi*^, affects both the anterior and posterior sides of the dorsal compartment (Fig 3I). Although Ci is only expressed in the anterior compartment in wild‐type discs, with *apGal* ^*ts*^ we are forcing the expression of a dominant negative construct in the entire dorsal domain. Therefore, Ci^DN^ is likely able to bind and repress its targets also in the posterior compartment, even though these genes are not normally regulated by Ci in the posterior.

We also perturbed the Hh pathway using a temperature‐sensitive allele of *hh* (*hh* ^*ts2*^), combined with a null mutant, *hh* ^*AC*^ (Lee *et al*, 1992; Ma *et al*, 1993). This genetic combination (*hh* ^*ts2*^/*hh* ^*AC*^) has been used previously in the literature to reduce Hh signaling upon temperature shift (Chang *et al*, 2013). At the permissive temperature (18°C), we observe a patterned response to OxPhos inhibition that is similar to that of wild type at 25°C: ATP declines slower in organizer regions (Appendix Fig S2A). After a 24 h shift to restrictive temperature, however, the patterned response is no longer visible (Appendix Fig S2B). To verify that the shift in temperature alone cannot reduce the patterned response to OxPhos inhibition, we shifted wild‐type larvae to 30°C and found that the organizer regions of the wing disc pouch still lose ATP slower than elsewhere in the pouch (Appendix Fig S2C). Thus, the loss of the pattern in the *hh* ^*ts2*^/*hh* ^*AC*^ discs is due to the loss of Hh activity.

Taken together, our results indicate that upon OxPhos inhibition, Hh signaling promotes glycolytic ATP production in the region just anterior to the AP boundary. Deciphering the underlying molecular mechanism is an important challenge for future work.

### ATP declines slower in organizer regions upon induction of hypoxia

**Figure 4:**
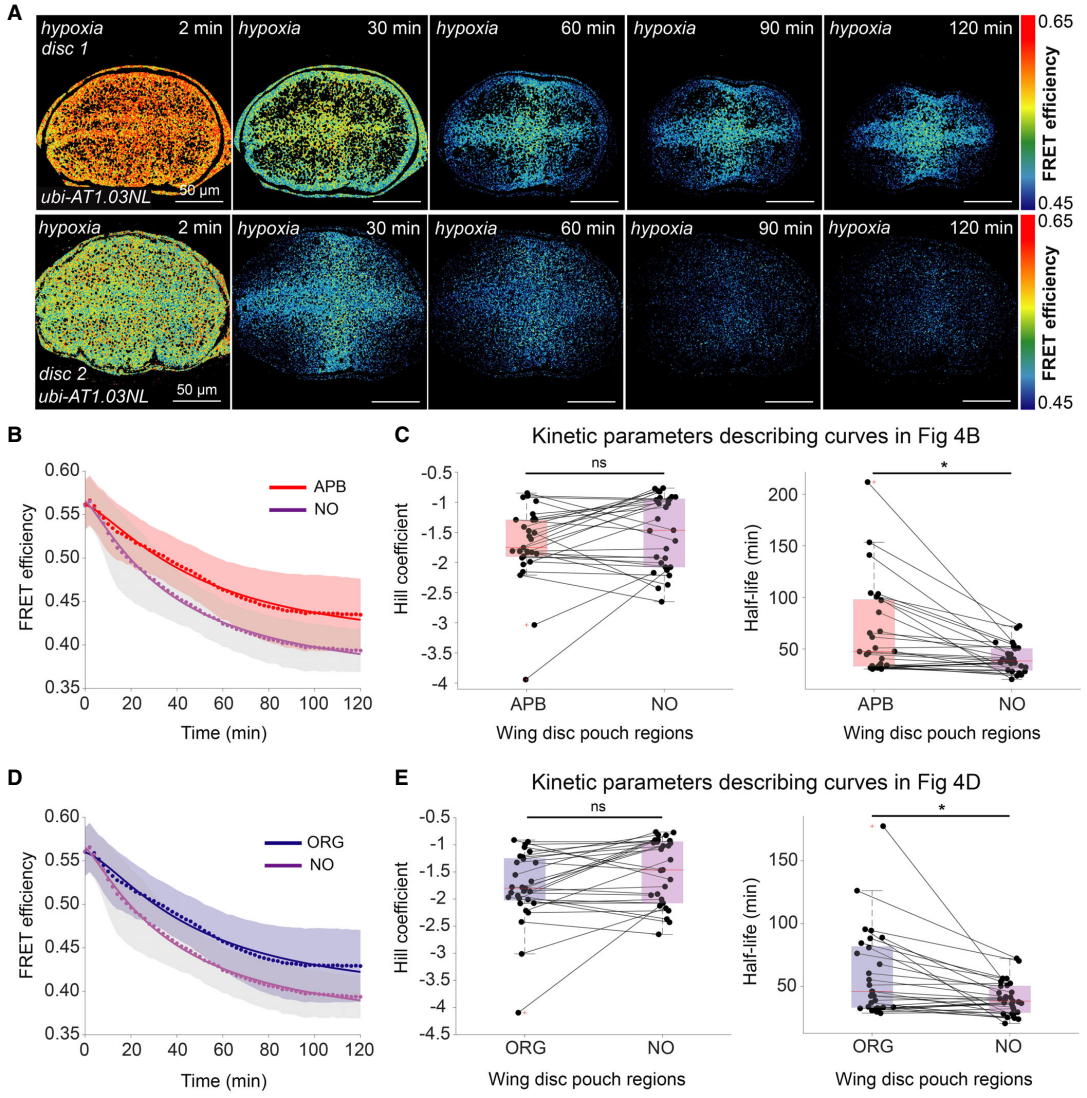
ATP declines slower in organizer regions of the wing disc pouch upon induction of hypoxia A
Timelapse montage of ATP sensor FRET efficiency upon induction of hypoxia in wing disc explants. To highlight the variability in this experiment, we show example discs from two separate experiments.B, D
Mean FRET efficiency measured over time in non‐organizers (NO) compared to (B) Anterior–Posterior boundary (APB) or (D) all organizer regions (ORG). Shaded regions indicate standard deviation; dots are the means per timepoint, and the solid lines indicate the fit to the mean.C, E
Kinetic parameters of the fit lines for ATP decline in (B) and (D). Lines connect the corresponding regions of the same disc; each dot represents data from one disc; box plots summarize the data: boxes encompass the 2^nd^–3^rd^ quartiles, with whiskers indicating the 1^st^ and 4^th^ quartiles and the red line indicating the median. ns = not significant *P*‐value *P‐value < 0.05 using a Kruskal–Wallis test (*n* = 29 discs).

During development, *Drosophila* larvae can encounter hypoxic conditions, as they burrow deep into their food source (Callier *et al*, 2015). To determine whether the same metabolic patterning occurs under realistic hypoxic conditions, we dynamically monitored ATP levels in the wing pouch of explants while reducing oxygen concentrations by continuously flowing nitrogen gas into the microscope chamber (Fig 4). Strikingly, we obtain similar results as with antimycin A (compare Figs 1C and 4A): ATP levels decline everywhere but slower at the organizer regions. Due to variability in the completeness of oxygen deprivation that we were able to achieve on the microscope, we observed variability in the final state in ATP levels of the tissue after 2 h (Fig 4A). Nonetheless, quantification of the kinetics indicates a significantly different half‐life in the organizer regions, in particular in the area just anterior to the AP boundary (the Hh signaling region), compared to the regions outside the organizers (Fig 4B–E)

In summary, examining the kinetics of ATP decline after inhibiting ATP production has uncovered spatial differences in ATP metabolism in the wing disc pouch. Organizers at compartment boundaries, which are areas of high morphogen signaling, appear to consume ATP slightly faster than the rest but also produce more ATP from glycolysis. Genetic perturbations support a role for Hh signaling in promoting ATP production from glycolysis. Such behavior could preferentially preserve energy in these important signaling centers during brief episodes of physiologically relevant energy stress, including hypoxia.

Importantly, future work will be needed to determine whether the observed differences in ATP metabolism between organizer and non‐organizer regions are only induced in response to OxPhos inhibition, or whether they are already present prior to the perturbation. Given that we see regional differences in ATP kinetics relatively quickly (< 30 min) during OxPhos inhibition, we favor the idea that the organizers are already primed for glycolysis more than other regions of the wing pouch. The slight but statistically significant differences in consumption rates (Fig 2B and C) could suggest that there is an increased energetic burden in the organizer regions from signaling itself. We propose that Hh may normally promote glycolytic ATP production to accommodate this increased energetic burden, allowing for rapid buffering of local energy fluctuations. The increased glycolytic capacity observed in the organizers during OxPhos inhibition may reflect this activity.

Glycolysis is especially thought to support the energy demands of maintaining the plasma membrane potential (Balaban & Bader, 1984; Lynch & Balaban, 1987; Epstein *et al*, 2014). In our previous paper, we saw that depletion of glycolytic enzymes by RNAi reduced steady‐state levels of ATP over long timescales, resulting in deregulated plasma membrane potential (Spannl *et al*, 2020). This energy stress and deregulated membrane potential actually increased Hh activity, indicating that Hh activity is partially suppressed under normal conditions. Thus, Hh can promote glycolysis during energy stress (shown in this work) but once restored, plasma membrane potential provides negative feedback on Hh signaling (shown in Spannl *et al*, 2020). Therefore, in contrast to the positive feedback noted for FGF/Wnt/glycolysis during vertebrate somatogenesis (Bulusu *et al*, 2017; Oginuma *et al*, 2017), we observe that the relationship between Hh and glycolysis in the wing disc is more of a homeostatic loop, which could stabilize the status quo, damping noise in either signaling or ATP production or both.

In conclusion, although significant attention has been paid to the role of glycolysis in promoting growth, as well as providing ATP and metabolites for rapid proliferation (Lunt & Vander Heiden, 2011; Abdel‐Haleem *et al*, 2017), our work suggests additional functions for glycolysis in preserving signaling during energy stress and promoting robustness of patterning and energy metabolism.

## Materials and Methods

### Fly stocks, husbandry, and genetics

The following fly stocks were used: Wild‐type Oregon‐R (BDSC #5), *ap‐Gal4* (BDSC #3041), *tub‐Gal80* ^*ts*^ (BDSC #7017 or 1019), *tub‐Gal4* (BDSC #5138), *UAS‐Ptc* ^*RNAi*^ (NIG #2411R‐1, after sequencing of its genomic DNA – see below), *UAS‐AT1.03RK* (a gift from T. Uemura, Graduate School of Biostudies, Kyoto University, Kyoto, Japan; Tsuyama *et al*, 2013) and *UAS‐Ci* ^*DN*^ (Ci^Cell^) (Méthot & Basler, 1999). All flies and larvae were raised on a standard food containing cornmeal, agar, malt, sugar beet syrup, brewery yeast, propionic acid, and soy flour under a 12 h light/dark cycle. All the knockdown and over‐expression experiments for immunostainings and FRET analysis were performed with *apGal4‐Gal80* ^*ts*^ (abbreviated here as *apGal* ^*ts*^ or *ap^ts^*). Twenty to thirty female *apGal* ^*ts*^ flies were crossed to male *UAS‐Ptc* ^*RNAi*^ or *UAS‐Ci* ^*DN*^ flies in a 3:1 ratio in a normal food vial. Flies were allowed to lay eggs in this vial in a 20°C incubator or water bath and then were transferred to a new vial every day. Larval growth took place at 20°C for 1 week, and then larvae were transferred to 30°C for 24 h. Then, upcrawling larvae were selected, and both sexes were dissected. In all cases, the necessary controls (outcrosses with wild‐type Oregon‐R flies) were handled in the same way. For Fig EV1F and G, *tubGal4* > *UAS‐AT1.03RK* larvae were raised in a 25°C incubator. For Appendix Fig S2, we introduced *ubi‐AT1.03NL* via recombination into the *hh* ^*ts2*^ flies (BDSC #1684, Ma *et al*, 1993). We also brought *hh* ^*AC*^ (BDSC #1749, Lee *et al*, 1992) over a TM6B balancer. Then we crossed the two lines, allowed their offspring larvae to grow at 18°C, and then moved them to 30°C for 24 h. Upcrawling larvae with the genotype *hh* ^*ts2*^, *ubi‐AT1.03NL/hh* ^*AC*^ were selected for the experiments. For controls, we also tested *ubi‐AT1.03NL* grown at 30°C, as well as *hh* ^*ts2*^, *ubi‐AT1.03NL/hh* ^*AC*^ grown exclusively at 18°C.

### Generation of transgenic lines

The FRET sensor ubi‐AT1.03NL was generated as described in (Spannl *et al*, 2020). Additionally, *apGal* ^*ts*^ was introduced into the background of the ubi‐AT1.03NL flies and was used for the spatial‐temporal overexpression of *Ptc* ^*RNAi*^ and *Ci* ^*DN*^.

### Imaging the FRET‐based ATP sensor in wing explants

Wing discs from upcrawling third‐instar larvae were dissected within 10 min in full medium: Grace's medium (Sigma G9771) supplemented with 5% FBS (ThermoFischer/Invitrogen 10270098) and 20 nM of 20‐hydroxyecdysone (Sigma H5142; Dye *et al*, 2017). They were then mounted as in (Spannl *et al*, 2020): basal side up on glass‐bottom dishes (MatTek Corporation, #P35G‐1.0‐20 C) with a double‐sided tape spacer and immobilized with a Whatman™ Cyclopore™ track‐etched polycarbonate membrane filter (GE Healthcare Life Sciences, #7062‐2513). Then, 1 ml of full medium was added, and samples were transferred to the microscope. All imaging experiments were performed at 25°C, including the ones with *Ptc* ^*RNAi*^, as we assume that new expression of Ptc will require longer than the 2 h experiment.

For the experiments with metabolic drugs, 1 ml of full medium with 2× concentration of antimycin A (Sigma‐Aldrich #A8674), 3‐bromopyruvate (Sigma Aldrich #16490) or 2‐deoxy‐D‐glucose (CARLROTH #CN96.3) was further added to the dish on the microscope using a hole on the lid. Drugs were added (shown with a black arrow in figures) either immediately after acquiring the first time point (time point 0 min) or after 10 min. Final concentrations are listed in the figure legends.

For the hypoxia experiments, the samples (in 1 ml of full medium) were placed in a well‐isolated chamber and nitrogen gas was flowed continuously from timepoint 1 min (initiation of flow) until the end of the experiments (25°C).

Images of the wing disc pouch were acquired on an Olympus IX81 microscope equipped with CSU‐W1 spinning disc (Yokogawa), Andor iXon Ultra 888, Monochrome EMCCD camera, Prior PRO SCAN III, Prior NanoScanZ, and an incubation chamber to ensure stable temperature (25°C). For all experiments, a 60× silicone oil immersion objective lens was used (UPLSAPO60xS2, NA = 1.3). Wing discs were excited with a 445 nm laser twice in a sequential manner. Emission of mse‐CFP was collected upon first excitation using an HQ 480/40 bandpass filter, and emission of cpVenus‐FRET was collected using an HQ 542/27 filter. The bleedthrough of mse‐CFP into the HQ 542/27 filter was estimated by exciting wing discs expressing *ubi‐Gal4*‐driven CFP‐tagged human cytoplasmic β‐actin (BDSC #7064) and acquiring images through an HQ 480/40 filter (*I*_*D*_) and its bleedthrough in an HQ 542/27 filter (*I*_*bth*_). The fraction of FRET intensity contributed by bleed‐through is given by the following equation:

$$\beta =\frac{I_{\mathrm{bth}}}{I_{D}}$$

### Analysis of FRET‐based ATP sensor imaging

FRET data were analyzed as previously described (Spannl *et al*, 2020). Briefly, a custom‐written MATLAB (MathWorks) script was used to estimate the FRET efficiency from the fluorescence images after smoothening both donor and FRET images using a 5 × 5 averaging kernel (Spannl *et al*, 2020). Donor (*I*_*D*_) and FRET (*I*_*F*_) images were background subtracted, and the FRET intensity was corrected for bleedthrough as:

$$I_{\text{FRET}}=I_{F}-\beta I_{D}$$

Finally, the FRET efficiency (*η*) was calculated as:

$$\eta =\frac{I_{\text{FRET}}}{I_{D}+I_{\text{FRET}}}$$

To separately quantify ATP dynamics in regions of the wing disc, the MATLAB script was modified by the Image Analysis Clinic of MPI‐CBG to calculate FRET efficiency in user‐defined circular ROIs of 20 μm diameter (Fig EV1I). Using the maximum intensity projection of the cpVenus channel (not the FRET), it is possible to discern AP and DV boundary regions by eye (Fig EV1H). The AP boundary appears as a stripe of slightly lower intensity, and the DV boundary lies in the middle of two stripes of higher intensity. Using the MATLAB script, we define circular nine ROIs (Fig EV1I). The organizer region of the AP boundary was defined as the average of three circular ROIs in the AP compartment boundary, whereas the organizer region of the DV boundary was defined as the average of three circular ROIs in the DV compartment boundary. AP and DV boundaries shared the central ROI. Consequently, the average FRET efficiency of both organizer regions was estimated from these five circular ROIs described in the AP and DV compartment boundaries. Non‐organizer regions were defined as the average of four circular ROIs laying outside the AP and DV compartment boundaries (Fig EV1I). To quantify FRET efficiency over the entire dorsal or ventral compartment of each disc (Figs 3H–K, EV3 and EV4A, B and E–H), a freehand tool was used to select these regions and calculate their average FRET efficiency.

### Empirical fit of ATP decline

To describe the kinetic data, we used a four‐parametric logistic curve that fits the data well. This fitting allows us to estimate the initial and final state of the FRET efficiency, the time needed to reach half of the FRET efficiency (half‐life, units of time), and the slope of the curve at the half‐life (Hill coefficient, unitless). To do so, data from the FRET efficiency decline over time upon addition of metabolic drugs were fit using MATLAB (R2021a, The MathWorks Inc.) with the following equation:

$$f\left(x\right)=\text{base}+\frac{\max\;-\;\text{base}}{1+{\left(\frac{t_{1/2}}{x}\right)}^{n}}$$

where $t_{1/2}$ and n are the two fit parameters corresponding to the half‐life (loss of 50% of FRET efficiency) and the Hill coefficient, respectively. Base and max were incorporated to account for the fact that our samples have variable starting and ending FRET values. The base was set to be 0.2827, as this is the lowest reliable recorded FRET efficiency value derived from the z‐stack of a morphologically healthy, unperturbed wing disc expressing ubiquitously the UAS‐AT1.03RK sensor using *tub‐Gal4* (Fig EV1C). Max is also a fit parameter that never exceeded 0.7, which is the highest reliable mean FRET efficiency value that we have recorded, derived from morphologically healthy, unperturbed wing discs expressing ubiquitously the AT1.03NL sensor. Graphs were generated from the same script.

We use the half‐life and Hill coefficient to compare the kinetics of ATP depletion in different tissue regions. Since the FRET efficiency decreases from a high state to a lower state, the Hill coefficient is negative. Consequently, a lower Hill coefficient corresponds to a steeper transition from the initial to the final state. In addition, lower values of half‐life correspond to faster kinetics of ATP depletion. We compare relative changes in these kinetic fit parameters across different regions of the tissue.

### Measurement of bulk ATP levels using a luciferase‐based assay

Bulk levels of ATP were measured using a luciferase‐based biochemical assay (ATPlite Luminescence Assay System, PerkinElmer). Wing discs from third instar upcrawling larvae were dissected in culture medium, washed with PBS within seconds, suspended in 20 μl of PBS, and added to wells of white polysterene flat bottom 96‐well assay plates (Costar® 3917) containing 80 μl of PBS (to make a final volume of 100 μl in PBS). Specifically, for Fig EV2A and B, experiments were done with paired discs from each larva (right and left). One disc was treated with either 3BP or antimycin A (Fig EV2A) or 3BP and 2DG (Fig EV2B) and the other was mock‐treated, serving as a control. The blank control was 100 μl of PBS. Samples were lysed by the addition of 50 μl of mammalian cell lysis solution followed by shaking at 700 rpm for 10 min. Luciferase substrate solution was added in a volume of 50 μl, and the samples were shaken at 700 rpm for 5 min. After 10 min of incubation in the dark, luciferase activity was measured using a Perkin Elmer Envision plate reader. To estimate concentration, a standard curve of luciferase activity was generated using a serial dilution of a 10 mM ATP stock solution.

To calculate the average wing disc volume, dissected wing discs from upcrawling larvae expressing the AT1.03NL FRET sensor were mounted and placed on the Olympus IX81 microscope as described earlier (Imaging FRET‐based ATP in wing explants). Wing discs were excited with a 445 nm laser, and emission of cpVenus‐FRET was collected using an HQ 542/27 filter. Serial acquisition of images every 0.5 μm from the most apical to the most basal disc part resulted in a z‐stack including the entire wing disc. The MPI‐CBG Image Analysis Clinic provided a FIJI macro that processes the z‐stacks based on the fluorescence intensity (emission collected using the HQ 542/27 filter) and calculates the wing disc volume. The volume of 14 wing discs was used to estimate the average wing disc volume. To estimate the intracellular ATP concentration, the amount of ATP measured from single‐disc luciferase assays was divided by the average wing disc volume.

### Immunofluorescence

Wing discs from upcrawling third instar larvae were dissected in PBS, fixed in 4% paraformaldehyde (PFA) for 20 min, and rinsed three times in PBS. Wing discs were then permeabilized with 0.05% Triton X‐100 in PBS (PBX) twice for 10 min, blocked for 45 min in PBX + 1 mg/ml BSA + 250 mM NaCl (BBX 250), and incubated overnight with the primary antibody in PBX + 1 mg/ml BSA (BBX) at 4°C. After washing twice for 20 min in BBX, wing discs were blocked for 45 min in the blocking solution (BBX + 4% normal goat serum) and incubated for 2–3 h with the secondary antibody in the blocking solution. Afterward, the wing discs were rinsed two times and washed three times within 45 min in PBX and the same in PBS. Finally, wing discs were mounted in VectaShield® (Vector Labs, #H‐1000). Secondary antibodies conjugated with Alexa Fluor® 488 and 555 were diluted 1:1000 and Alexa Fluor® 647 were diluted 1:500 (ThermoFisher Scientific). Primary antibodies used:

**Table:** 

Antigen	Host species	Dilution used	Origin	Genotype
Ptc	Mouse	1:100	DSHB, AB_528441	*apGal* ^*ts*^ > *Ptc* ^*RNAi*^/*WT*
Ci_155_	Rat	1:30	DSHB, AB_2109711	*apGal* ^*ts*^ > *Ptc* ^*RNAi*^/*WT*
PH3	Rabbit	1:500	Cell signaling #9701S	*apGal* ^*ts*^ > *Ptc* ^*RNAi*^/*WT*

Images were acquired using a Zeiss LSM700 inverted confocal microscope equipped with a Zeiss Axio Observer.Z1, a motorized stage Maerzhauser Wetzler Gmbh EK 130 × 85 mot. Tango CZ EMV, a 25×/0.8 LCI Plan‐Neofluor, W/Glyc/Oil objective (Zeiss), and 2 PMT. Both samples and controls were dissected, fixed, stained, and imaged in parallel so that the reagents and handling conditions were always the same. Fiji (Schindelin *et al*, 2012) was used for image processing, orienting, and segmenting. Z‐stacks of PH3 were max‐projected and segmented using the Weka segmentation plugin (Arganda‐Carreras *et al*, 2017) as previously described (Dye *et al*, 2017). Statistical analyses and plots were made using GraphPad Prism 9.

To confirm the colocalization of the FRET pattern close to the AP boundary with the Ptc stripe (Fig EV1J and K), FRET efficiency was calculated in wing discs while treated with 10 μM antimycin A for up to 60 min, as previously described (Imaging FRET‐based ATP in wing disc explant). At the end of 60 min, the wing discs were fixed on site with 4% PFA, and the glass bottom plates with the samples were removed so that immunostainings for the detection of Ptc with Alexa Fluor® 647 could continue, as described above. At the end of the immunostainings, the samples were taken back to the spinning disc microscope. Wing discs were excited with a 638 nm laser, and emission of Alexa Fluor® 647 was collected with a HQ 685/40 filter. Afterward, the same wing discs were compared in the FRET and immunofluorescence images (Fig EV1J and K).

### Statistical analyses

Statistical analyses were performed using GraphPad Prism 9 or MATLAB (R2021a, The MathWorks Inc.). For statistical significance, Kruskal–Wallis, paired *t*‐tests, Mann–Whitney tests, one‐way ANOVA with Bonferroni correction, and unpaired *t*‐tests with Welch's correction were performed as listed in the figure legends for each experiment. The use of either parametric or non‐parametric statistical analysis was determined by the normal or not normal distribution of data, respectively. Number of biological replicates for each experiment is stated in the figure legends. We only excluded data from wing discs in which some of the regions were no longer visible by the end of the experiment, for example, if the sample moved considerably or if a bubble in the immersion oil occluded the sample.

### Note on Gal4 lines

In control experiments with Gal4 driver lines alone (no UAS construct), we found that ATP levels dropped more uniformly throughout the wing pouch upon antimycin A addition (compare Appendix Fig S1A–H with Fig 1C–G). This result demonstrates that care should be taken with these genetic tools, as either the expression of the Gal4 itself or the disruption of gene function due to the insertion can subtly alter energy metabolism in a way that is detectable with this sensitive method for monitoring ATP dynamics. Here, we use *apGal* ^*ts*^ so that we have an internal control (ventral compartment), and we used strong gain‐of‐function perturbations, which should dominate any metabolic effects caused by the Gal4 or differences in genetic background.

### Bioinformatic analysis of whole‐genome sequence of Ptc^RNAi^ fly line

Because we were unsure of the origin of the Ptc^RNAi^ line, we sequenced its genome. Isolated genomic DNA from the Ptc^RNAi^ fly line was analyzed by GENEWIZ Germany GmbH (NGS short‐read WGS). Illumina reads were filtered for adapter sequences and low‐quality bases by using Trim Galore (version 0.6.6, https://zenodo.org/badge/latestdoi/62039322). The remaining 158 million read pairs (323X average coverage) were mapped with bwa mem (version 0.7.17‐r1198, default arguments; Li & Durbin, 2009) to the reference genome (NCBI accession: GCF_000001215.4). A manual inspection of the alignments revealed that exon 2 of the *Ptc* gene had two times higher read coverage compared to the remaining parts (exons + introns) of the *Ptc* gene (312X and 946X, respectively).

The Illumina reads were also assembled with the *de novo* assembler Spades (version 3.15.4, Prjibelski *et al*, 2020) providing the additional arguments ‐‐isolate and ‐‐trusted‐contigs GCF_000001215.fasta. The assembled 126,190 contigs (N50: 169 Kb, size: 161 Mb) were aligned to the reference genome with minimap2 (version: 2.24‐r1122, Li, 2021). A manual inspection showed that the *Ptc* gene region in chromosome 2R was fully covered by a single contig (Node_334). Additionally, two secondary local alignments of one contig (Node_420) only covered the exon 2 region of *Ptc*. The primary alignment of contig Node_420 maps to chromosome 3R [12,866,885–12,980,960] almost front to end, where the first 2,996 bp of contig Node_420 do not agree with the reference chromosome 3R and were soft‐clipped from the alignment.

The exon 2 sequence of *Ptc* was mapped with blastn (version 2.11.0 Altschul *et al*, 1990) against the complete *de novo* assembly, which confirmed the three mapping positions. First, the original position corresponding to 2R: Node_334 [108,449–108,942], and second, two alignments corresponding to the integration into 3R: Node_420 [1,433–942] and [1,814–2,305].

Illumina reads were aligned to the *de novo* assembly with bwa. The read coverage profile of contig Node_420 [1–2,600] lies in 280–330X, which is in agreement with the average overall coverage and indicates two separate *Ptc* exon 2 off‐target integration sites in chromosome 3R.

## Author contributions

**Ioannis Nellas:** Conceptualization; data curation; formal analysis; validation; investigation; visualization; writing – original draft; writing – review and editing. **K Venkatesan Iyer:** Software; formal analysis; methodology; writing – review and editing. **Juan M Iglesias‐Artola:** Software; formal analysis; investigation; visualization; methodology; writing – review and editing. **Martin Pippel:** Formal analysis. **André Nadler:** Formal analysis; supervision; funding acquisition; writing – original draft; project administration; writing – review and editing. **Suzanne Eaton:** Conceptualization; supervision; funding acquisition; project administration. **Natalie A Dye:** Data curation; supervision; writing – original draft; project administration; writing – review and editing.

In addition to the CRediT author contributions listed above, the contributions in detail are:

IN performed experiments and analyzed data. KVI established the FRET acquisition and analysis methods, and JMIA developed the algorithmic tools used by IN to fit and analyze the FRET data. MP performed the bioinformatic analysis of the *Ptc* ^*RNAi*^ fly line. SE conceived the study and supervised early work on the project. IN, SE, NAD, and AN designed the study. NAD and AN directed the project after the death of SE. IN and NAD wrote the manuscript with discussions and feedback from all authors.

## Disclosure and competing interests statement

The authors declare that they have no conflict of interest.

## Supporting information



Appendix

Expanded View Figures PDF

PDF+

## Data Availability

Source data for the figures have been deposited into Edmond, the Open Research Data Repository of the Max Planck Society: https://doi.org/10.17617/3.WXFFCN.
