# Supplementary material for: Hedgehog signaling can enhance glycolytic ATP production in the Drosophila wing disc
Source: EMBO Rep. 2022 Sep 22;23(11):e54025. doi: 10.15252/embr.202154025 (PMC9638854; doi:10.15252/embr.202154025)
Supplement: Supplementary file 1 — Appendix [file EMBR-23-e54025-s002.pdf]

Appendix: Nellas et al

Table of Contents:

Appendix Figure S1 ……………………………2

Appendix Figure S2 ……………………………3

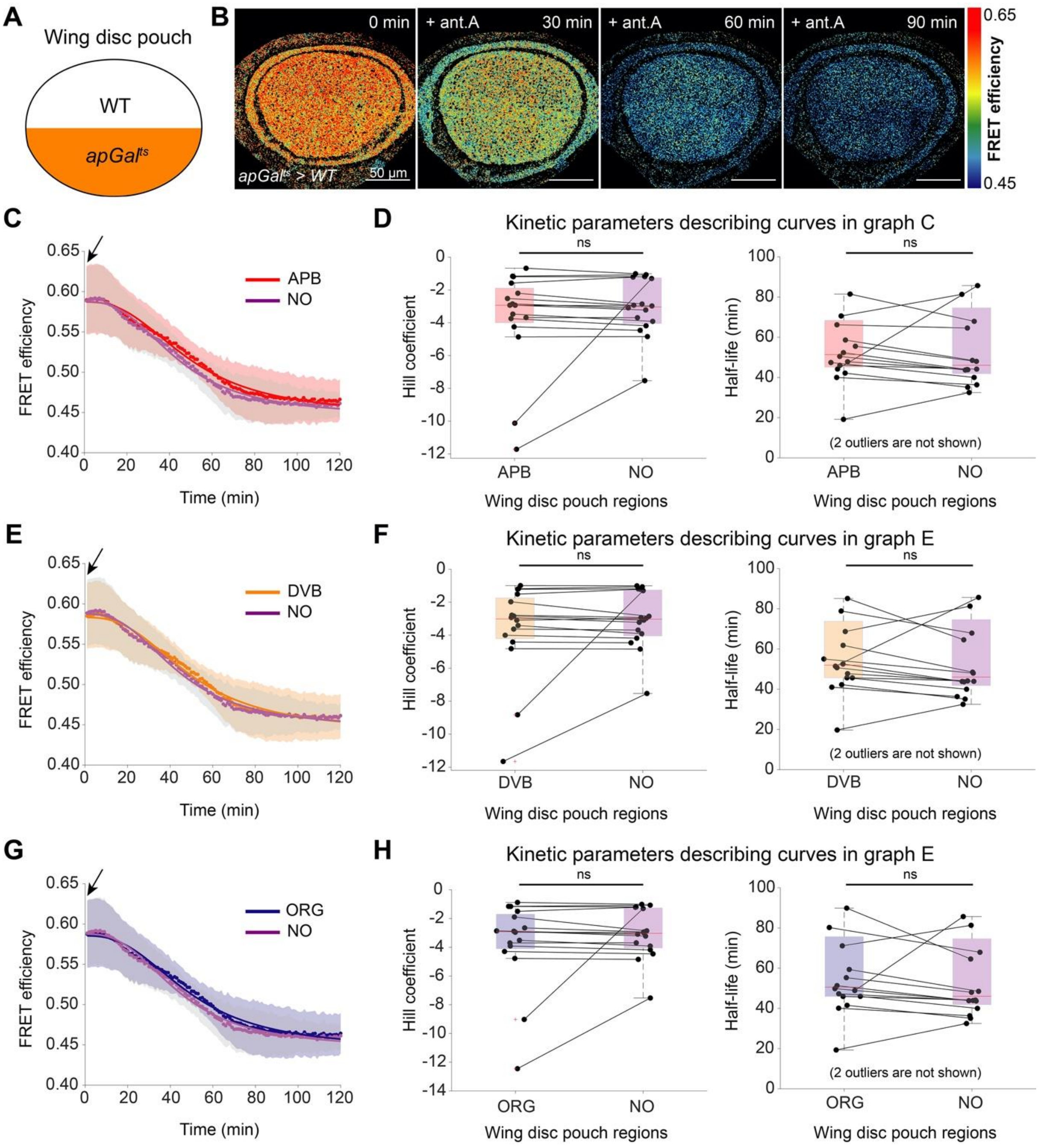

Appendix Figure S1: Extended regional analysis of kinetics of ATP decline upon OxPhos inhibition in Fig EV3 *(apGalts>WT)*. (A) Schematic representation of *apGal*^{ts} expression in the dorsal compartment; ventral compartment serves as an internal control. (B) Timelapse montage of ATP sensor FRET efficiency in the wing pouch after 10 μM antimycin A (ant.A) addition in *apGal*^{ts}>WT wing discs. (C) Timelapse of ATP sensor FRET efficiency across the wing pouch after addition of 10 μM ant.A. (C, E, G) Mean FRET efficiency measured over time in the (C) AP boundary (APB), (E) DV boundary (DVB), or (G) both AP and DV boundaries (ORG=organizers) and non-organizer regions (NO). Shaded regions indicate standard deviation (SD); filled circles indicate mean per timepoint, and solid lines indicate the fit to the mean. Black arrows indicate the addition of the drug. (D, F, H) Fit parameters of individual time traces for AP boundary and non-organizer regions (NO) (D), DV boundary and non-organizer regions (NO) (F), and total organizers (ORG) and non-organizer (NO) regions (H). Each dot represents data from one disc, and black lines connect the corresponding regions of the same disc. Box plots summarize the data: boxes encompass the 2nd-3rd quartiles, with whiskers indicating the 1st and 4th quartiles and the red line indicating the median. ns = not significant p-value using a Kruskal-Wallis test (n=16).

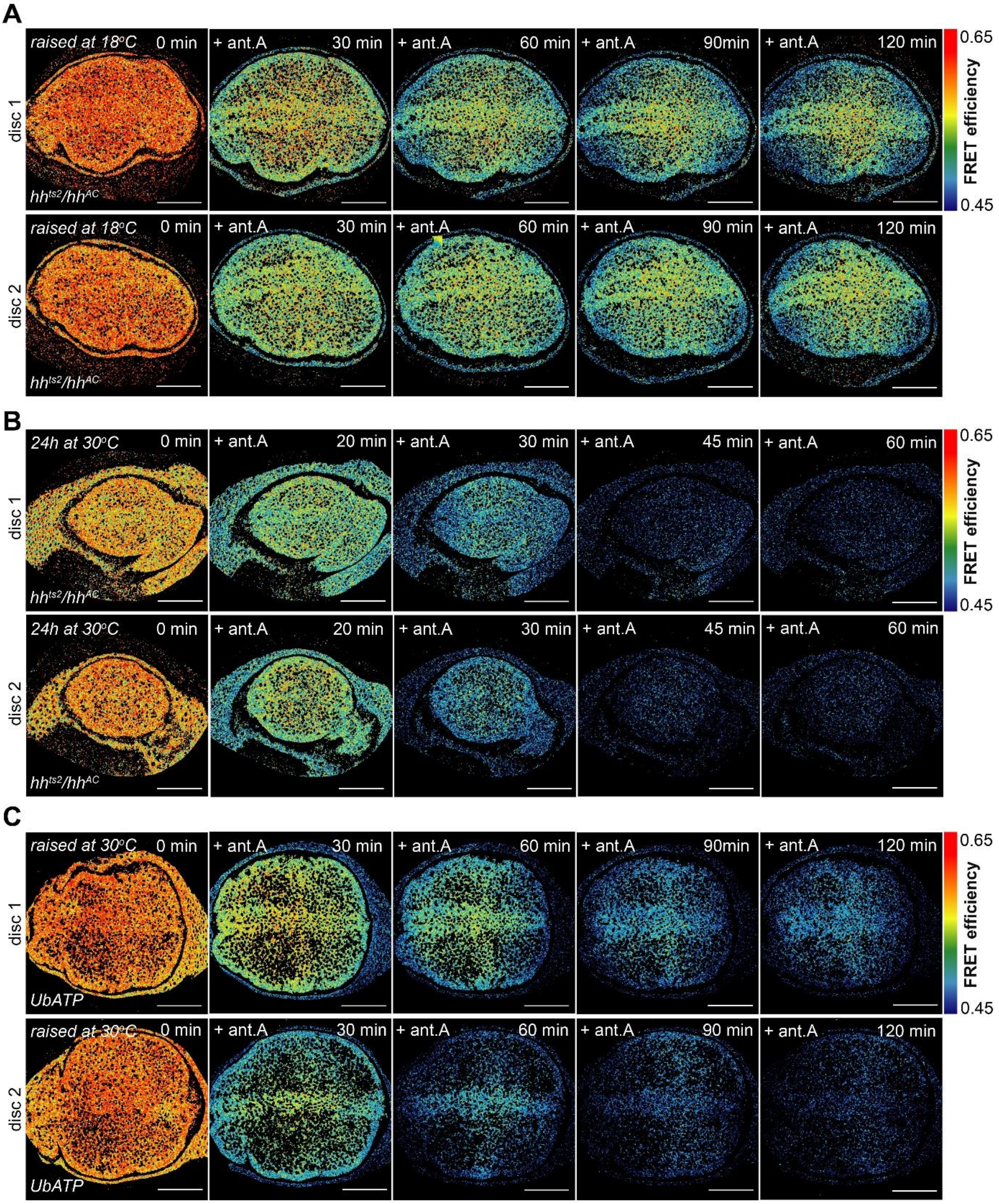

Appendix Figure S2. Loss of Hh protein activity reduces patterned response to OxPhos inhibition. (A) Timelapse montages of ATP sensor FRET efficiency in wing disc explants from *hh*^{ts2}/*hh*^{AC} larvae raised at permissive temperature (18°C) phenocopy the patterned response to OxPhos inhibition in wild type (Fig 1). (B) Timelapse montages of ATP sensor FRET efficiency in wing disc explants from *hh*^{ts2}/*hh*^{AC} larvae shifted to the restrictive temperature (30°C) do not show a patterned response to OxPhos inhibition (representatives from 13 discs from 3 independent experiments). Note that the wing pouch is also smaller when grown at restrictive temperature, consistent with the loss of Hh pathway. (C) Timelapse montages of ATP sensor FRET efficiency in wing disc explants from wild type UbATP larvae raised at 30°C demonstrate that the patterned response to OxPhos inhibition persists after shift to 30°C. Scale bars indicate 50 µm.
