# Supplementary material for: Hedgehog signaling can enhance glycolytic ATP production in the Drosophila wing disc
Source: EMBO Rep. 2022 Sep 22;23(11):e54025. doi: 10.15252/embr.202154025 (PMC9638854; doi:10.15252/embr.202154025)
Supplement: Supplementary file 2 — Expanded View Figures PDF [file EMBR-23-e54025-s004.pdf]

Expanded View Figures

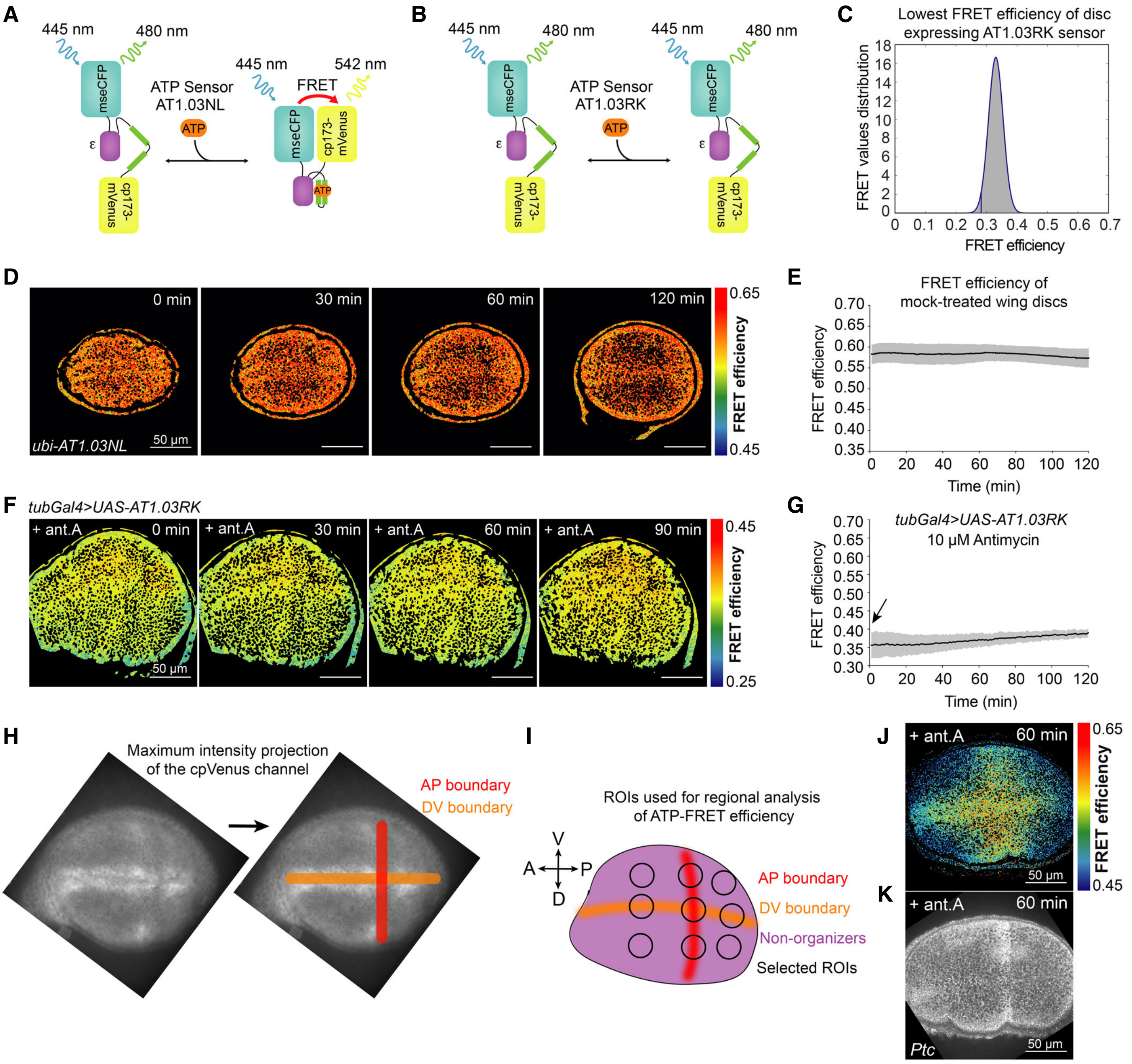

Figure EV1.

 © 2022 The Authors

◀ **Figure EV1. ATP levels in the wing disc pouch are spatially uniform and stable in culture without OxPhos inhibition.**

A, B Schematic of ATP-FRET sensor (AT1.03NL, A) and its ATP-insensitive version (AT1.03RK, B).

C Histogram showing the distribution of FRET efficiency values across a 70-plane z-stack of a morphologically healthy, unperturbed wing disc ubiquitously expressing the AT1.03RK sensor: gray area corresponds to the FRET values, and the blue curved line shows a Gaussian fit. The lowest FRET efficiency value used for data fitting was 0.2827 (blue vertical line), defined as the mean FRET efficiency minus twice the standard deviation (SD).

D Timelapse montage of ATP-FRET sensor efficiency in the wing disc pouch during culture for 2 h without any drugs.

E Mean FRET efficiency in the entire pouch measured over time; gray shade indicates SD, and small black dots represent the means per timepoint ($n = 9$).

F Timelapse montage of FRET efficiency in the wing disc pouch expressing the ATP-insensitive construct (AT1.03RK) upon addition of 10 μM antimycin A (ant.A).

G Mean FRET efficiency in the entire pouch in (F). Black arrow indicates the addition of the drug; gray shade indicates SD, and small black dots represent the means per timepoint ($n = 9$).

H Anterior-posterior (AP) and Dorsal-ventral (DV) boundary regions are discernible with a maximum intensity projection of the cpVenus channel. The AP boundary appears as a stripe of slightly lower intensity, and the DV boundary lies in the middle of two stripes of higher intensity.

I Schematic indicating the location of the ROIs that were used to calculate mean FRET efficiency in different wing pouch regions. The three ROIs in the AP boundary or in the DV boundary were averaged together to calculate the mean FRET efficiency of the AP boundary and DV boundary, respectively. The organizer region was measured as the average of all five of these ROIs (AP + DV boundaries). The non-organizer region corresponds to the remaining four ROIs outside of the AP + DV boundaries. To verify that the region of slower kinetics of ATP loss upon antimycin addition corresponds to the Hh signaling domain, we fixed discs in the middle of antimycin treatment (after 60 min) and stained for Patched (Ptc).

J Spatial pattern of FRET efficiency after 60 min of 10 μM antimycin A (ant.A) exposure.

K Ptc expression in the same disc as (J) after fixation and immunofluorescence.

© 2022 The Authors

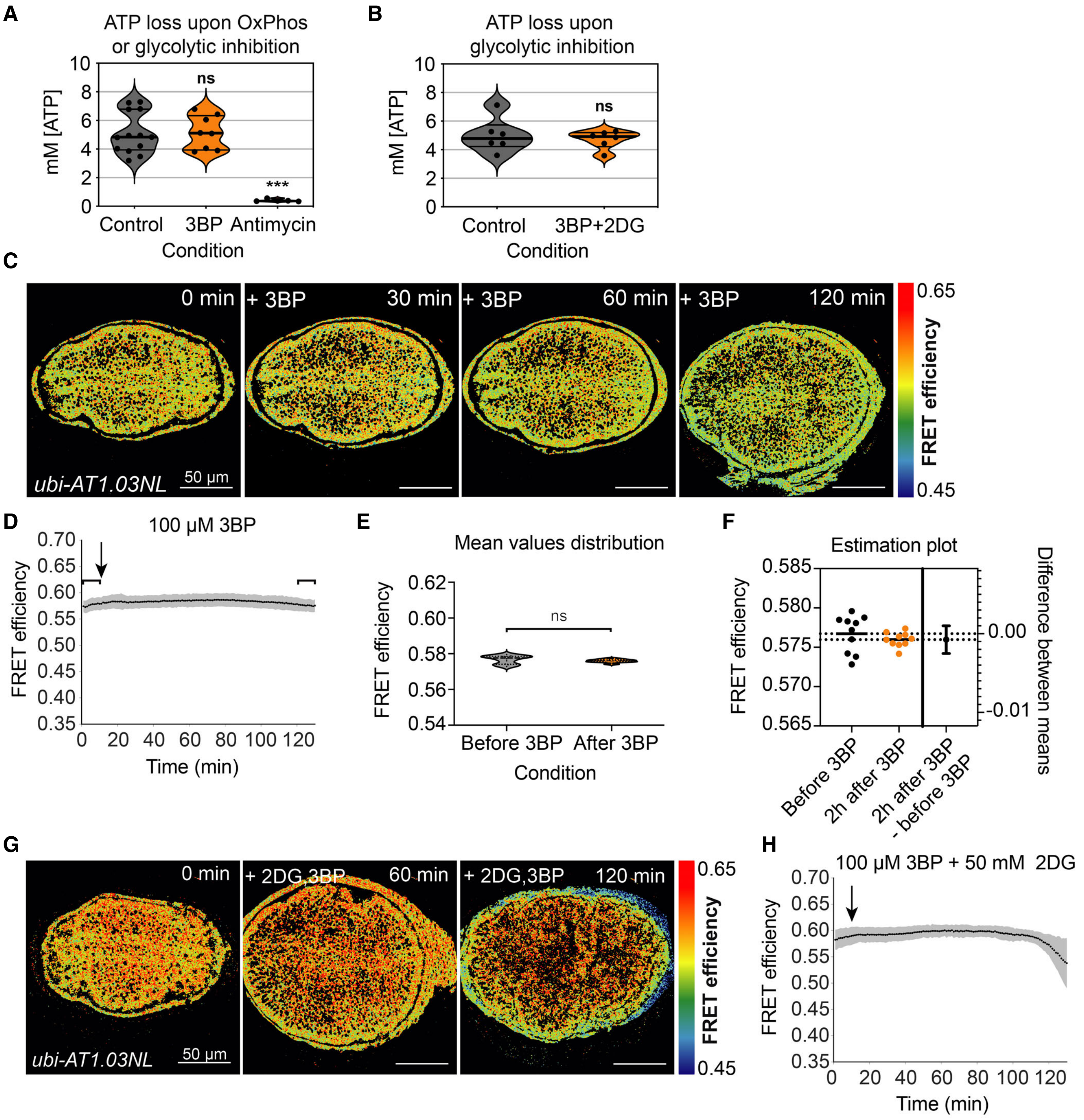

Figure EV2.

 © 2022 The Authors

Figure EV2. Glycolysis inhibitors alone do not significantly affect ATP levels.

A ATP levels measured using a luminescence-based biochemical assay from single discs after a 2 h treatment with either 50 μM 3-bromopyruvate (3BP) (n = 8) or 10 μM antimycin A (n = 5) compared to untreated control discs (n = 13). ***P-value < 0.001, ns = not significant using either Mann–Whitney test (control vs. 3BP) or unpaired t-test (control vs. antimycin A).

B ATP levels of single discs after 1 h of treatment with 50 μM 3BP + 50 mM 2-deoxy-D-glucose (2DG) (n = 6) compared to untreated control discs (n = 6). ns = not significant P-value using a paired t-test. In (A) and (B), the frequency distributions of the data are shown in violin plots, with the thick black horizontal line indicating the median and the thinner lines indicating the quartiles.

C Timelapse montage of ATP-FRET sensor efficiency after 3BP addition.

D–F Mean FRET efficiency time trace of the entire wing pouch; shaded region indicates standard deviation (SD); black arrows indicate the addition of the drug. Brackets indicate the mean FRET values before and 2 h after 3BP addition (1–10 and 121–130 min, respectively). The distribution of mean values within these brackets are shown as violin plots in (E) (solid line indicates median, dotted indicate quartiles). These were compared using an unpaired t-test (ns = not significant P-value, n = 11 discs) including (F) Welch's correction and estimation plot (n = 11 discs for each group). In (F), horizontal dotted lines run through the mean values for the two samples (before 3BP and 2 h after 3BP); on the right, the difference between the two means is plotted on a separate axis (right), where the bars indicate the upper and lower 95% confidence intervals.

G Timelapse montage of ATP-FRET sensor efficiency after 3BP + 2DG addition.

H Mean FRET efficiency measured over time in the entire wing disc pouch upon addition of 2DG + 3BP. Black line indicates the mean, and gray shade indicates SD (n = 9). Black arrows indicate the addition of the drugs.

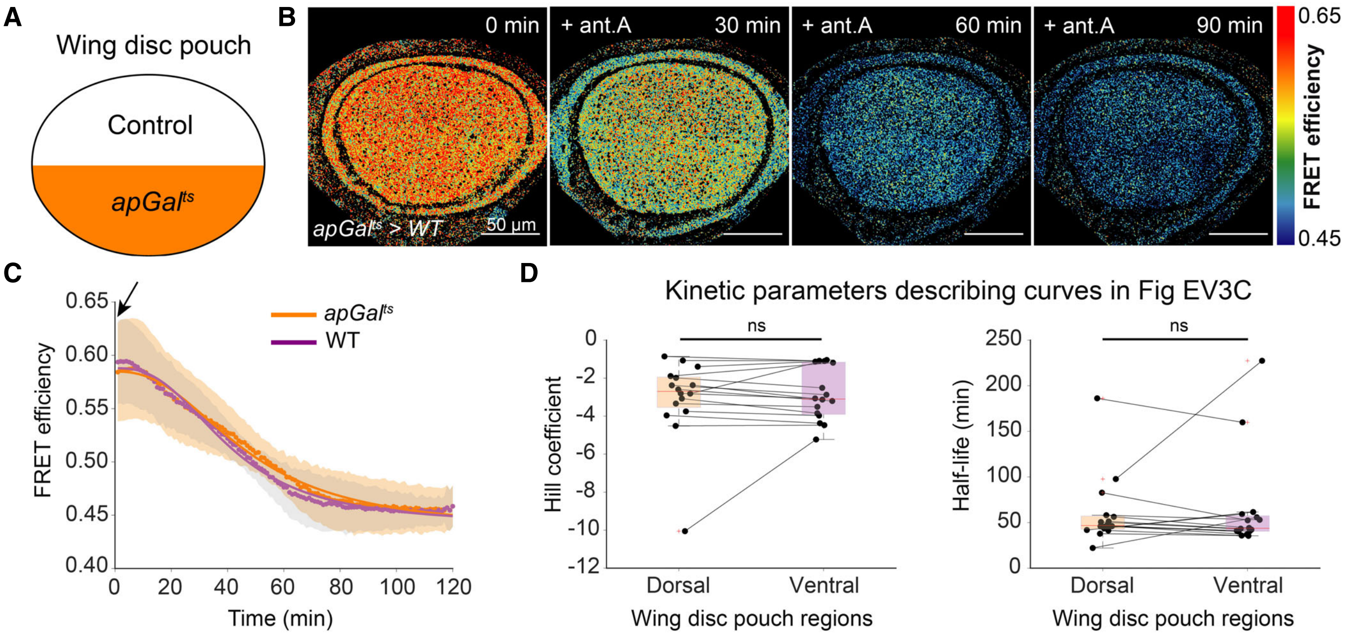

Figure EV3. ATP levels decline with similar kinetics in the dorsal and ventral compartments of the *apGal^{ts}* genetic background (without a UAS construct).

A Schematic representation of *apGal^{ts}* expression in the dorsal compartment; ventral compartment serves as an internal control.

B Timelapse montage of ATP sensor FRET efficiency in the wing disc pouch after 10 μM antimycin A (ant.A) addition in *apGal^{ts}* > WT wing discs.

C Mean FRET efficiency measured over time in the dorsal and ventral compartments. Shaded regions indicate standard deviation; dots are the means per timepoint, and solid lines illustrate the fit to the mean.

D Fit parameters of individual time traces for dorsal and ventral compartments shown in (C). Each dot represents data from one disc, and lines connect the corresponding regions of the same disc. Box plots summarize the data: boxes encompass the 2nd–3rd quartiles, with whiskers indicating the 1st and 4th quartiles and the red line indicating the median. ns = not significant P-value, using Kruskal–Wallis test (n = 16). Black arrow indicates the addition of the drug.

© 2022 The Authors

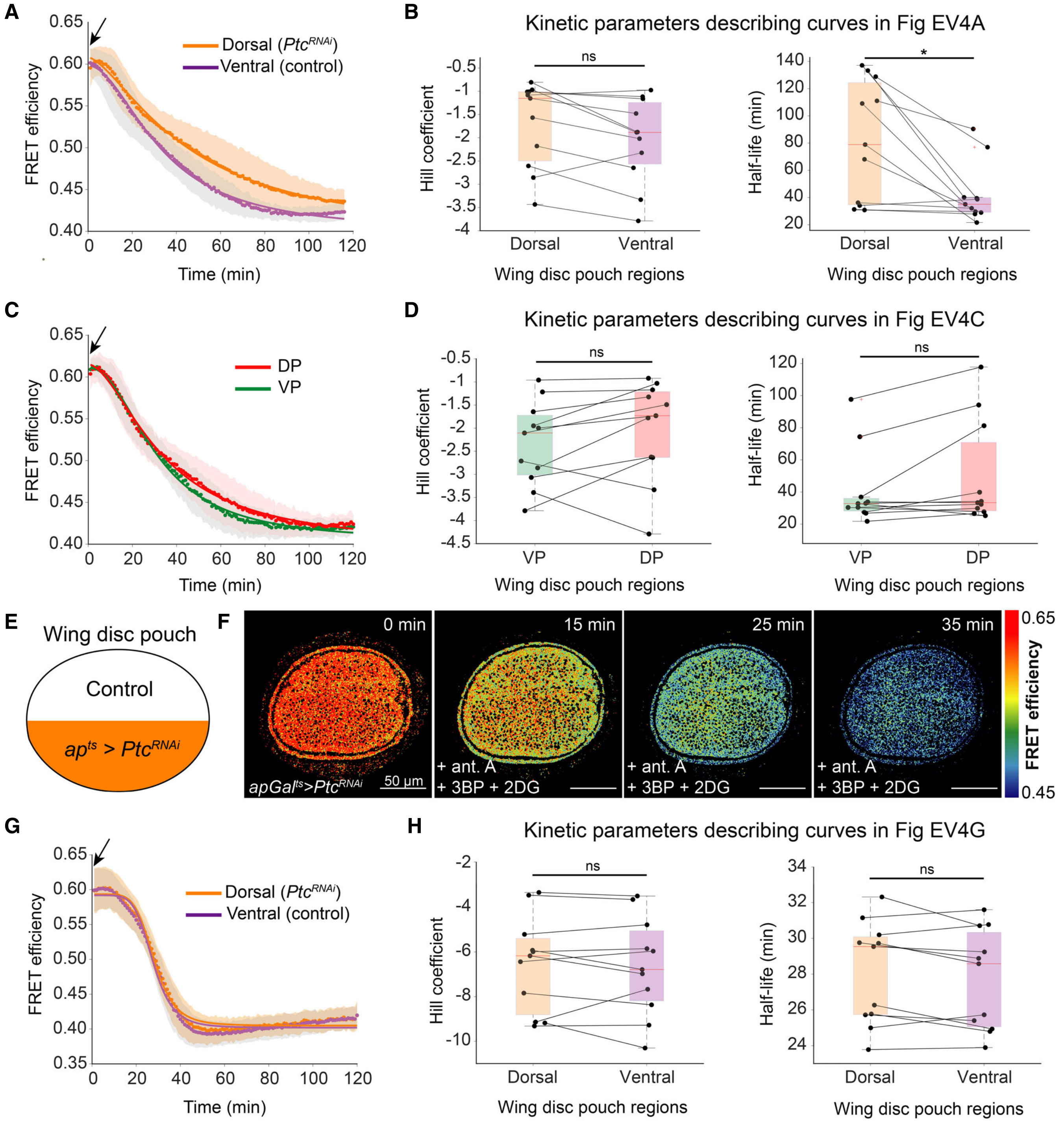

Figure EV4.

© 2022 The Authors

Figure EV4. Extended regional analysis of *Ptc^{RNAi}* upon OxPhos inhibition alone or combined with glycolysis inhibition.

A, C Mean FRET efficiency measured over time in the dorsal and ventral compartments (A) or the ventral posterior (VP) and dorsal posterior (DP) sub-compartments (C) of the wing disc pouch in *apGal^{ts}* > *Ptc^{RNAi}* upon OxPhos inhibition. Shaded regions indicate standard deviation (SD); dots are the means per timepoint, and solid lines illustrate a fit to the mean; black arrow indicates the addition of drug.

B, D Fit parameters of individual time traces for the dorsal and ventral compartments (B) or the posterior sub-compartments (D). Each dot represents data from one disc, and lines connect the corresponding regions of the same disc. Box plots summarize the data: boxes encompass the 2nd–3rd quartiles, with whiskers indicating the 1st and 4th quartiles and the red line indicating the median. **P*-value < 0.05, ns = not significant *P*-value, using a Kruskal–Wallis test (n = 11).

E Schematic representation of *apGal^{ts}* > *Ptc^{RNAi}* expression in the dorsal compartment.

F Timelapse montage of ATP-FRET sensor efficiency after addition of antimycin A (ant.A), 3-bromopyruvate (3BP), and 2-deoxy-D-glucose (2DG).

G Mean FRET efficiency measured over time in the dorsal and ventral compartments. Shaded regions indicate SD; dots are the means per timepoint, and solid lines illustrate a fit to the mean data; black arrow indicates the addition of drugs.

H Fit parameters of individual time traces for dorsal and ventral compartments. Each dot represents data from one disc, and lines connect the corresponding regions of the same disc. Box plots summarize the data (see Materials and Methods). ns = not significant *P*-value, using Kruskal–Wallis test (n = 12).

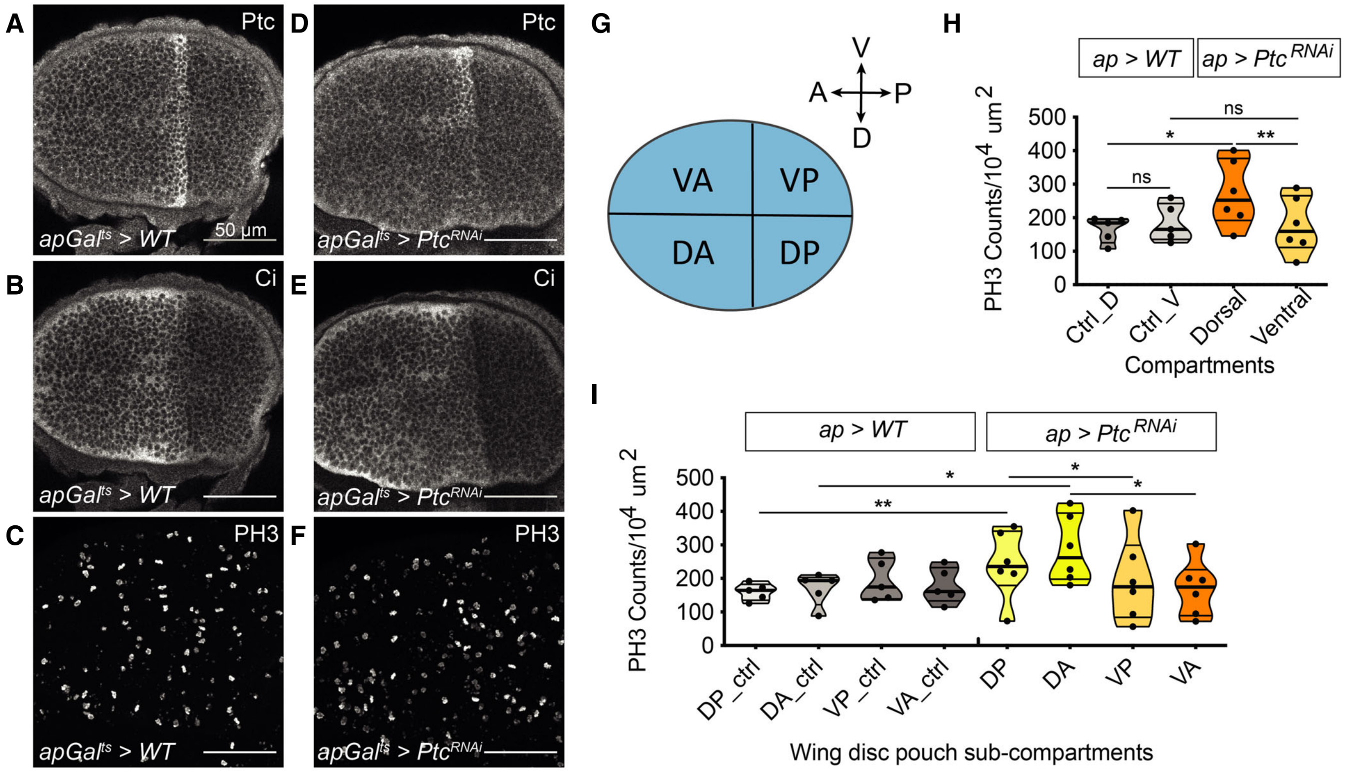

Figure EV5. Upregulation of Hh pathway activity with *Ptc^{RNAi}* increases proliferation in both anterior and posterior compartments.

A–F Control (*apGal^{ts}* > *WT*, A–C) or *apGal^{ts}* > *Ptc^{RNAi}* (D–F) wing pouch stained for Ptc (A, D), Ci (B, E) or the mitotic marker phospho-histone H3 (PH3, C, F).

G Schematic of wing disc sub-compartments. V = Ventral, D = Dorsal, A = Anterior, P = Posterior.

H Quantification of PH3-positive nuclei in the dorsal (D) and ventral (V) compartments of the pouch of control (Ctrl, *apGal^{ts}* > *WT*, n = 5) and *apGal^{ts}* > *Ptc^{RNAi}* (n = 6) wing discs. **P*-value < 0.05, ***P*-value < 0.01 using, ns = not significant *P*-value using either paired t-tests between disc compartments belonging to the same group (*apGal^{ts}* > *Ptc^{RNAi}* or control) or Mann–Whitney tests for different groups (Ctrl_D vs. Dorsal, Ctrl_V vs. Ventral). The frequency distributions of the data are shown with violin plots, with the thick black horizontal line indicating the median and the thinner lines indicating the quartiles.

I Quantification of mitotic density in different compartments of the pouch in control (Ctrl, *ap* > *WT*) and *apGal^{ts}* > *Ptc^{RNAi}* wing discs. **P*-value < 0.05, ***P*-value < 0.01 using, ns = not significant *P*-value using one-way ANOVA tests with Bonferroni *post hoc* correction.

© 2022 The Authors
