# Supplementary material for: Hedgehog signaling can enhance glycolytic ATP production in the Drosophila wing disc
Source: EMBO Rep. 2022 Sep 22;23(11):e54025. doi: 10.15252/embr.202154025 (PMC9638854; doi:10.15252/embr.202154025)
Supplement: Supplementary file 4 [file EMBR-23-e54025-s001.pdf]

EMBO reports

Hedgehog signaling can enhance glycolytic ATP production in the Drosophila wing disc

Ioannis Nellas, K. Venkatesan Iyer, Juan Iglesias-Artola, Martin Pippel, André Nadler, Suzanne Eaton, and Natalie Dye
DOI: 10.15252/embr.202154025

Corresponding author(s): Natalie Dye (natalie_anne.dye@tu-dresden.de)

Review Timeline:

Submission Date:	20th Sep 21
Editorial Decision:	14th Oct 21
Revision Received:	31st Mar 22
Editorial Decision:	29th Apr 22
Revision Received:	2nd Aug 22
Accepted:	22nd Aug 22

Editor: Martina Rembold/Deniz Senyilmaz-Tiebe

Transaction Report:

(Note: With the exception of the correction of typographical or spelling errors that could be a source of ambiguity, letters and reports are not edited. Depending on transfer agreements, referee reports obtained elsewhere may or may not be included in this compilation. Referee reports are anonymous unless the Referee chooses to sign their reports.)

Dear Dr. Dye,

Thank you for the submission of your research manuscript to our journal, which was now seen by two referees, whose reports are copied below.

Referees express interest in the role of Hedgehog signaling in regulation of glycolytic flux in the wing disc. However, they also raise important concerns that need to be addressed to consider publication here.

I find the reports informed and constructive, and believe that addressing the concerns raised will significantly strengthen the manuscript. As the reports are below, and I think all points need to be addressed, I will not detail them here.

Given these positive recommendations, we would like to invite you to revise your manuscript with the understanding that the referee concerns (as in their reports) must be fully addressed and their suggestions taken on board. Please address all referee concerns in a complete point-by-point response. Acceptance of the manuscript will depend on a positive outcome of a second round of review. It is EMBO reports policy to allow a single round of revision only and acceptance or rejection of the manuscript will therefore depend on the completeness of your responses included in the next, final version of the manuscript.

We generally allow three months as standard revision time. As a matter of policy, competing manuscripts published during this period will not negatively impact on our assessment of the conceptual advance presented by your study. However, we request that you contact the editor as soon as possible upon publication of any related work, to discuss how to proceed. Should you foresee a problem in meeting this three-month deadline, please let us know in advance and we may be able to grant an extension.

*** Temporary update to EMBO Press scooping protection policy:
We are aware that many laboratories cannot function at full efficiency during the current COVID-19/SARS-CoV-2 pandemic and have therefore extended our 'scooping protection policy' to cover the period required for a full revision to address the experimental issues highlighted in the editorial decision letter. Please contact the scientific editor handling your manuscript to discuss a revision plan should you need additional time, and also if you see a paper with related content published elsewhere.***

IMPORTANT NOTE: we perform an initial quality control of all revised manuscripts before re-review. Your manuscript will FAIL this control and the handling will be DELAYED if the following APPLIES:
1. A data availability section providing access to data deposited in public databases is missing (where applicable).
2. Your manuscript contains statistics and error bars based on n=2. Please use scatter plots in these cases.

You can submit the revision either as a Scientific Report or as a Research Article. For Scientific Reports, the revised manuscript can contain up to 5 main figures and 5 Expanded View figures. If the revision leads to a manuscript with more than 5 main figures it will be published as a Research Article. In this case the Results and Discussion section should be separate. If a Scientific Report is submitted, these sections have to be combined. This will help to shorten the manuscript text by eliminating some redundancy that is inevitable when discussing the same experiments twice. In either case, all materials and methods should be included in the main manuscript file

Supplementary/additional data: The Expanded View format, which will be displayed in the main HTML of the paper in a collapsible format, has replaced the Supplementary information. You can submit up to 5 images as Expanded View. Please follow the nomenclature Figure EV1, Figure EV2 etc. The figure legend for these should be included in the main manuscript document file in a section called Expanded View Figure Legends after the main Figure Legends section. Additional Supplementary material should be supplied as a single pdf labeled Appendix. The Appendix includes a table of content on the first page with page numbers, all figures and their legends. Please follow the nomenclature Appendix Figure Sx throughout the text and also label the figures according to this nomenclature. For more details please refer to our guide to authors.

Please note that for all articles published beginning 1 July 2020, the EMBO Reports reference style will change to the Harvard style for all article types. Details and examples are provided at
https://www.embopress.org/page/journal/14693178/authorguide#referencesformat

When submitting your revised manuscript, please carefully review the instructions that follow below. Failure to include requested items will delay the evaluation of your revision.

1) a .docx formatted version of the manuscript text (including legends for main figures, EV figures and tables). Please make sure that the changes are highlighted to be clearly visible.

2) individual production quality figure files as .eps, .tif, .jpg (one file per figure).

3) a .docx formatted letter INCLUDING the reviewers' reports and your detailed point-by-point responses to their comments. As

part of the EMBO Press transparent editorial process, the point-by-point response is part of the Review Process File (RPF), which will be published alongside your paper. For more details on our Transparent Editorial Process, please visit our website: https://www.embopress.org/page/journal/14693178/authorguide#transparentprocess
You are able to opt out of this by letting the editorial office know (emboreports@embo.org). If you do opt out, the Review Process File link will point to the following statement: "No Review Process File is available with this article, as the authors have chosen not to make the review process public in this case."

4) a complete author checklist, which you can download from our author guidelines (<http://embor.embopress.org/authorguide>). Please insert information in the checklist that is also reflected in the manuscript. The completed author checklist will also be part of the RPF.

5) Please note that all corresponding authors are required to supply an ORCID ID for their name upon submission of a revised manuscript (<https://orcid.org/>). Please find instructions on how to link your ORCID ID to your account in our manuscript tracking system in our Author guidelines (<http://embor.embopress.org/authorguide>).

6) We replaced Supplementary Information with Expanded View (EV) Figures and Tables that are collapsible/expandable online. A maximum of 5 EV Figures can be typeset. EV Figures should be cited as 'Figure EV1, Figure EV2" etc... in the text and their respective legends should be included in the main text after the legends of regular figures.

- For the figures that you do NOT wish to display as Expanded View figures, they should be bundled together with their legends in a single PDF file called *Appendix*, which should start with a short Table of Content. Appendix figures should be referred to in the main text as: "Appendix Figure S1, Appendix Figure S2" etc. See detailed instructions regarding expanded view here: <http://embor.embopress.org/authorguide#expandedview>.

- Additional Tables/Datasets should be labeled and referred to as Table EV1, Dataset EV1, etc. Legends have to be provided in a separate tab in case of .xls files. Alternatively, the legend can be supplied as a separate text file (README) and zipped together with the Table/Dataset file.

7) We would also encourage you to include the source data for figure panels that show essential data.

Numerical data should be provided as individual .xls or .csv files (including a tab describing the data). For blots or microscopy, uncropped images should be submitted (using a zip archive if multiple images need to be supplied for one panel). Additional information on source data and instruction on how to label the files are available <http://embor.embopress.org/authorguide#sourcedata>.

8) Our journal encourages inclusion of *data citations in the reference list* to directly cite datasets that were re-used and obtained from public databases. Data citations in the article text are distinct from normal bibliographical citations and should directly link to the database records from which the data can be accessed. In the main text, data citations are formatted as follows: "Data ref: Smith et al, 2001" or "Data ref: NCBI Sequence Read Archive PRJNA342805, 2017". In the Reference list, data citations must be labeled with "[DATASET]". A data reference must provide the database name, accession number/identifiers and a resolvable link to the landing page from which the data can be accessed at the end of the reference. Further instructions are available at <http://embor.embopress.org/authorguide#datacitation>.

9) Please make sure to include a Data Availability Section before submitting your revision - if it is not applicable, make a statement that no data were deposited in a public database. Primary datasets (and computer code, where appropriate) produced in this study need to be deposited in an appropriate public database (see <http://embor.embopress.org/authorguide#dataavailability>).

Please remember to provide a reviewer password if the datasets are not yet public.

The accession numbers and database should be listed in a formal "Data Availability " section (placed after Materials & Method) that follows the model below. Please note that the Data Availability Section is restricted to new primary data that are part of this study.

Data availability

The datasets (and computer code) produced in this study are available in the following databases:

- RNA-Seq data: Gene Expression Omnibus GSE46843 (https://www.ncbi.nlm.nih.gov/geo/query/acc.cgi?acc=GSE46843)
- [data type]: [name of the resource] [accession number/identifier/doi] ([URL or identifiers.org/DATABASE:ACCESSION])

*** Note - All links should resolve to a page where the data can be accessed. ***

10) Regarding data quantification, please ensure to specify the name of the statistical test used to generate error bars and P values, the number (n) of independent experiments underlying each data point (not replicate measures of one sample), and the test used to calculate p-values in each figure legend. Discussion of statistical methodology can be reported in the materials and methods section, but figure legends should contain a basic description of n, P and the test applied.
Please note that error bars and statistical comparisons may only be applied to data obtained from at least three independent biological replicates.
Please also include scale bars in all microscopy images.

We would also welcome the submission of cover suggestions, or motifs to be used by our Graphics Illustrator in designing a cover.

I look forward to seeing a revised version of your manuscript when it is ready. Please let me know if you have questions or comments regarding the revision.

Yours sincerely,

Deniz Senyilmaz Tiebe

Deniz Senyilmaz Tiebe, PhD
Editor
EMBO Reports

Referee #1:

Summary

Using a previously described FRET-Based ATP sensor the authors started by showing that Drosophila wing pouch have uniform steady state ATP levels. However, upon chemical inhibition of OxPhos, spatially heterogeneity is revealed in the wing pouch with the wing discs' organizer regions resisting more to ATP drop than the remaining tissue.
Next, the authors investigate whether glycolysis-derived ATP production could justify the slower drop of ATP in the organizer regions. By pharmacological inhibition of glycolysis+OxPhos they show that spatially heterogeneity is abolished hence stating that organizer regions have enhanced glycolysis, at least upon OxPhos inhibition, compared to the non-organizer ones.
Then, by genetically interfering with the Hh pathway, authors show that repression or activation of the Hh pathway alters the impact of OxPhos inhibition in ATP levels in the organizer regions. Moreover, knock-down of Patched softens the ATP drop in the wing pouch upon OxPhos inhibition. The same is not observed when both glycolysis and OxPhos are inhibited. These results led the authors to postulate that Hh signaling has a role in the increase in glycolysis-derived ATP observed in the wing disc organizer upon OxPhos inhibition.

Major comments

The authors show that the wing disc organizers have a different metabolic behavior upon OxPhos inhibition, which seems to involve Hh signaling. Although this is well described, several times throughout the article the authors generalize the role of Hh in regulating Glycolysis in the entire disc, which is not supported by their data. We recommend for the authors to revise the text to correct these issues.

Fig 3C - Why is there not a slower ATP decay in the VA/VP boundary/organizer? (compared to Fig. 1) As Ptc is only affecting the Dorsal side, and the authors have not referred that this RNAi affects organizer formation in general, the anterior side of the organizer should behave as in WT+Ant only.

Fig 3C - Why is the overall ATP level decay in Fig.3C slower then in Fig.1? This different rate can be seen when comparing the ventral sides of these discs (yellow vs dark blue).

Minor comments
Line 82. hindered glycolysis upregulates Hh signaling - This was shown in the other paper. This should read something like "this work and previous..."
Section 1
Line 99. These areas are known growth "organizer" regions, with high signaling activity in the Wingless/Notch and Hedgehog pathways, respectively. - add a reference here
Extended data S1I - Why is the FRET efficiency after 60 min in S1I so different from the one in Fig1C?

Extended data S1J - This image is not well explained in the main text.
Figure 1E-F and HI - Authors should state what is represented by the error bars and shaded regions.
Section 2
Line 139. What is the difference between these 2 inhibitors? Why combining it to block glycolysis? Add the rationale behind it to the text.

Fig. 2C-D - Authors should state what is represented by the error bars.

Section 3
Fig S3B - 'Shaded regions indicate SD; solid lines illustrate a fit of the mean data.' This information should be found in S3C legend instead.
FigS3D-E - Authors should state what is represented by the error bars.
Fig 3E-F, H-I - Authors should state what is represented by the error bars.

Section 4
Fig4D-E - Authors should state what is represented by the error bars.
- Authors talked about the insensitive control of the ATP biosensor and showed the range of FRET-values obtained with this tool. However, it would be good to have an image of a wing disc expressing ATP [RKRK] upon inhibition of glycolysis to confirm that no differences between OR and NO regions are observed.

- Since authors suggested that glycolysis upregulation in the organizer region prevent a faster decline in ATP level, it would be interesting to see whether downregulation of glycolytic enzyme genes alters the pattern observed upon OxPhos inhibition (not required).

Materials and Methods

Line 294. UAS-PtcRNAi (BDSC) - Authors should refer stock number

Referee #2:

In this paper, Nellas et al explore the relationship between Hh signalling and ATP metabolism, using the Drosophila wing as a model. The Eaton group had previously shown with a FRET sensor that ATP levels modulate Hh signaling (Spannl, 2020). Here the authors report a reverse effect. Although ATP levels are relatively uniform in wing imaginal discs, relatively higher ATP is detected at the D/V and A/P boundaries upon inhibition of OxPhos. The focus of the present paper is on the residual ATP detected at the A/P boundary. From the result of double inhibition experiments, the authors suggest that residual ATP is caused by higher glycolysis. They then manipulate Hh signalling to test whether increased residual ATP at the A/P boundary is a consequence of increased Hh signalling there. They conclude that Hh signalling stimulates aerobic glycolysis, and that this could be part of a homeostatic mechanism since ATP levels have been suggested to boost Hh signalling activity.

The basic phenomenon described in this paper is somewhat contrived (a genetic effect is only seen in a situation of pharmacological inhibition). However, it can be argued that this is the way to uncover unsuspected activities. Nevertheless, the significance of the observation remains limited because, even if Hh signalling boosts glycolysis, this must be a relatively minor phenomenon since higher ATP levels cannot be detected in untreated tissues. It is conceivable that any increase in glycolysis is compensated by a reduction in OxPhos (implying a different metabolic program at the organisers) but this is not considered by the authors.

The authors conclude from the double inhibition experiments that glycolysis is higher at the organisers than elsewhere. However, one cannot exclude the possibility that these cells could be particularly resistant to Oxphos inhibition or that inhibition of Oxphos leads to differential changes in ATP turnover.

I do not understand the effect of CiDN in the P compartment. There is no Hh signaling in this compartment so it is not clear how further inhibiting Hh signalling would have an effect, unless, as the authors imply, this is due to a neomorphic activity of CiDN. In this case, an alternative means of inhibiting Hh signalling (smo RNAi?) is needed.

It is not clear what the authors mean by a homeostatic mechanism as a way to link the present study to the previous publication of this group.

As it stands this manuscript reports an interesting observation but fails to properly document the underlying mechanism or its wider significance.

Additional comment.

In fig. 3C, the Ptc RNAi effect seems to spread into P compartment. Is this real?

Referee #3:

In this manuscript, Nellas, et al follow up on recent work from the lab (Spanni, et al) to assess the impact of altering glucose metabolism mechanisms on ATP generation in the Drosophila wing disc and define how the Hedgehog signaling pathway contributes to the process. Using a FRET sensor for ATP, the authors report differential impact of oxidative phosphorylation inhibition in "organizer" regions at the anterior-posterior (AP) and dorsal-ventral (DV) boundaries of the wing disc. Slower decay of FRET is observed in the organizer regions, suggesting that ATP is retained for longer than in "non-organizer" cells. Doubly inhibiting oxidative phosphorylation and glycolysis eliminates the delayed response, supporting a model in which glycolysis remains active in organizer regions for longer after inhibition of oxidative phosphorylation. Extending recently published work, the authors demonstrate increased FRET upon constitutive activation of Hedgehog signaling (ptc knockdown) and accelerated FRET loss upon overexpression of a dominant negative form of Ci.

Strengths of the work include quantitative measurements of ATP levels in real time and space in a developing tissue, a technically challenging, but critically important problem. The question of the developmental roles and signaling impacts of carbohydrate metabolism is also of high importance, with this work addressing how inhibition of the major pathway (OxPhos) impacts the minor (glycolysis). The study complements the lab's recently published work that identified glycolysis as a regulator of Hh signaling amplitude via promoting Smo retention at the plasma membrane. Here, the reverse process is assessed, with measurement of the impact of Hh signaling on ATP generation via glycolysis. The two papers together support the existence of a negative feedback loop, whereby Hh signaling activates glycolysis and then glycolysis limits Hh signaling through regulation of plasma membrane potential and entry of lipid-based Hh pathway inhibitors. Here, the impact of Hh signaling on ATP production is clearly demonstrated and the links to glycolysis are reasonably well supported using drug inhibition studies.

The manuscript could benefit from addition of a biologically relevant context for the reported studies. For example, there are a number of ways that oxidative phosphorylation might be inhibited during larval development. Discussion of how Hh-mediated induction of glycolysis in organizer cells upon natural depletion of ATP via OxPhos inhibition (oxygen deprivation, starvation, etc) might impact wing disc cells and tissue and what the implications might if the mechanism is conserved in other Hh-dependent cells would provide a stronger justification for the relevance of the study for understanding the biology. This may require tightening up of the Introduction and Results to remain within the character limits, but would really provide a strong framework for the study and its implications.

The nature of FRET experiments is that they are variable in living tissues, and sensitivity can be a challenge. As such, the differences between conditions are small throughout the manuscript. In addition, glycolysis appears to be more of a back-up plan for the tissue than the primary energy production machine, adding to the relatively small effects. Placement of the work into a context of how this might be relevant in vivo, as suggested above, would increase the impact of the findings. Of note, the extensive statistical analysis largely mitigates the magnitude issue.

It would be helpful to have figures labeled more clearly with genotype and drug status. For example, it takes jumping back and forth from Figure 3 to the text to determine that 3E is untreated, ptc KD and 3F is antimycin A-treated, ptc KD. This is an issue throughout the figures. Labeling the figure itself or making the legend more clear would be helpful.

Nellas et al: Response to Reviewer comments

We thank all the reviewers for their insightful and relevant feedback on our manuscript. Addressing these comments has significantly improved the manuscript. To summarize the major changes:

1. All three reviewers raised concerns about the physiological relevance of our findings, in particular because a large part of our data relied on pharmacological inhibition.
   - We have taken this feedback seriously and designed an experiment to inhibit OxPhos by depleting oxygen. Our new results are consistent with our original experiments using antimycin A. We consolidated the Hh perturbations into one figure (now Fig 3) and include the new oxygen deprivation experiment in Fig 4.
2. Another common comment was that we did not sufficiently explain the significance of our work, how it fits with our previous data and what are the broader implications.
   - To address this problem, we considerably reworked the introduction to better motivate and frame our study, and we added paragraphs to the end of the manuscript to discuss our findings in more depth.
3. Reviewer 1 raised concerns about the variability of response to OxPhos inhibition, specifically that different images throughout the manuscript did not seem consistent.
   - To be sure that we are adequately describing the response, we significantly increased the N with additional experiments. Fig 1 has been changed to add these new data. The results have not changed, but there are now more replicates for this highly variable dataset. We also extended the time of analysis in this experiment to 120min, rather than 90min in the original paper, in order to make all the analyses throughout the paper consistent.

All additions to the text are highlighted in blue in the revised manuscript.

Referee #1:

Summary

Using a previously described FRET-Based ATP sensor the authors started by showing that Drosophila wing pouch have uniform steady state ATP levels. However, upon chemical inhibition of OxPhos, spatially heterogeneity is revealed in the wing pouch with the wing discs' organizer regions resisting more to ATP drop than the remaining tissue. Next, the authors investigate whether glycolysis-derived ATP production could justify the slower drop of ATP in the organizer regions. By pharmacological inhibition of glycolysis+OxPhos they show that spatially heterogeneity is abolished hence stating that organizer regions have enhanced glycolysis, at least upon OxPhos inhibition, compared to the non-organizer ones.

Then, by genetically interfering with the Hh pathway, authors show that repression or activation of the Hh pathway alters the impact of OxPhos inhibition in ATP levels in the organizer regions. Moreover, knock-down of Patched softens the ATP drop in the wing pouch upon OxPhos inhibition. The same is not observed when both glycolysis and OxPhos are inhibited. These results led the authors to postulate that Hh signaling has a role in the increase in glycolysis-derived ATP observed in the wing disc organizer upon OxPhos inhibition.

Response: We thank the reviewer for the careful feedback on our work. Below, we provide a point-by-point response to the specific issues raised.

Major comments

1. The authors show that the wing disc organizers have a different metabolic behavior upon OxPhos inhibition, which seems to involve Hh signaling. Although this is well described, several times throughout the article the authors generalize the role of Hh in regulating Glycolysis in the entire disc, which is not supported by their data. We recommend for the authors to revise the text to correct these issues.

Response: The reviewer is correct, and we have now made efforts to correct the text accordingly, for example lines 176-7. We have also significantly changed the introduction and discussion of results to better explain our interpretations of the data and its wider significance, in response to other reviewer comments. This revised version better clarifies how we think Hh and glycolysis are linked. All textual revisions are highlighted in blue in the revised manuscript.

2. Fig 3C - Why is there not a slower ATP decay in the VA/VP boundary/organizer? (compared to Fig. 1) As Ptc is only affecting the Dorsal side, and the authors have not referred that this RNAi affects organizer formation in general, the anterior side of the organizer should behave as in WT+Ant only.

Response: The reviewer is correct: ideally, the ventral side of the AptTS>PtcRNAi discs should resemble the wild type and thus show a region of slower kinetics anterior of the AP boundary (Fig 1). We do not observe this behavior, however. Importantly, we do not see the same metabolic activity of the organizers in the *apGal*^{ts} outcrossed to wild type (Fig EV3, Appendix figure 1): in this genetic background, the loss of ATP-FRET upon antimycin addition is the same everywhere. We tested the possibility that the temperature shift from 25C to 29C could have this effect, however we did not see any change in the pattern of ATP-FRET upon antimycin addition either when shifting wild type flies to 29C or outcrossed *apGal*^{ts} flies to 25C. We therefore conclude that the *apGal*^{ts} background itself somehow slightly alters ATP metabolism so that decline of ATP levels upon OxPhos inhibition happens more uniformly across the wing pouch. In fact, we also tested other Gal4 lines and observe the same effect with nubGal4, gal80ts (*nubGal*^{ts} , Reviewer Figure 1) and to a lesser extent with engrailedGal4 (not shown).

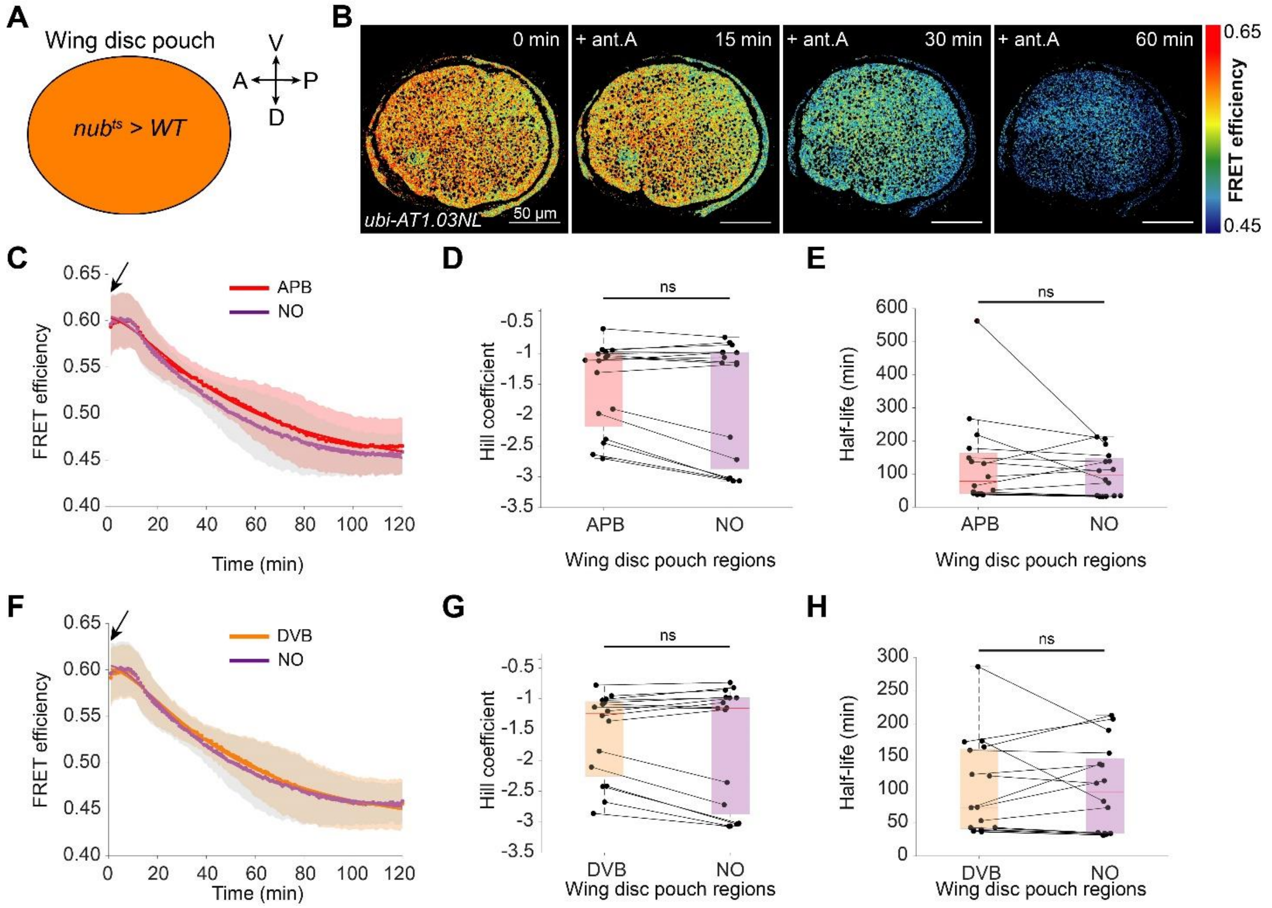

Reviewer Figure 1: NubGal4 does not show a patterned response to OxPhos inhibition (A) Schematic of *nubGal*^{ts} expression pattern (entire pouch). (B) Timelapse of ATP sensor FRET efficiency across the wing pouch after addition of 10 µM antimycin A (ant.A). (C, F) Mean FRET efficiency measured over time in the (C) AP boundary (APB) or (F) DV boundary (DVB) and non-organizer regions (NO). Shaded regions indicate standard

deviation (SD); filled circles indicate mean per timepoint, and solid lines indicate the fit to the mean. Black arrows indicate the addition of the drug. (D-E, G-H) Fit parameters of individual time traces for AP boundary and non-organizer regions (D-E) or DV boundary and non-organizer regions (G-H). Each dot represents data from one disc, and black lines connect the corresponding regions of the same disc. Box plots summarize the data (see Methods); ns = not significant p-value using a Kruskal-Wallis test (n=15).

Thus, we suspect that the issue lies not just in the specific insertion but with the exogenous expression of Gal4 itself. In the revision, we have added a note in the methods to explain these observations (lines 470-80). This is a potentially interesting result that perhaps indicates that the forced expression of this exogenous protein places extra energetic burden on the wing disc and they accommodate – however, this topic is beyond the scope of this report. In this manuscript, we limit ourselves to the *apGal*^{ts} construct so that we can use the ventral compartment as an internal control. Both the Ptc^{RNAi} and the Ci^{DN} perturbations are strong enough to overcome the effect of the Gal4 and show strong phenotypes in response to antimycin when comparing the perturbed dorsal compartment with the internal control of the ventral compartment. Thus, these experiments still provide strong evidence that the Hh pathway can affect energy production via glycolysis.

3. Fig 3C - Why is the overall ATP level decay in Fig.3C slower than in Fig.1? This different rate can be seen when comparing the ventral sides of these discs (yellow vs dark blue).

Response: There is considerable variation, both in the starting ATP levels and in the kinetics of ATP loss upon Antimycin addition, between individual discs and experiments. Thus, comparisons by eye in individual images can be misleading, and throughout the paper, we rely on quantitative comparisons of the kinetics subjected to statistical tests. Note that for the revision, we have actually provided additional replicates for Fig 1 and extended the time of analysis to 120min after the addition of drug (the results do not change but there is higher N). In Reviewer Figure 2, we directly compare our quantification of the kinetics of ATP decline upon OxPhos inhibition in the ventral anterior regions of the pouch highlighted by the reviewer: UbATP (wild type) vs the unperturbed, control compartment of the *apGal*^{ts} >*Ptc*^{RNAi} discs. There is no statistically significant difference.

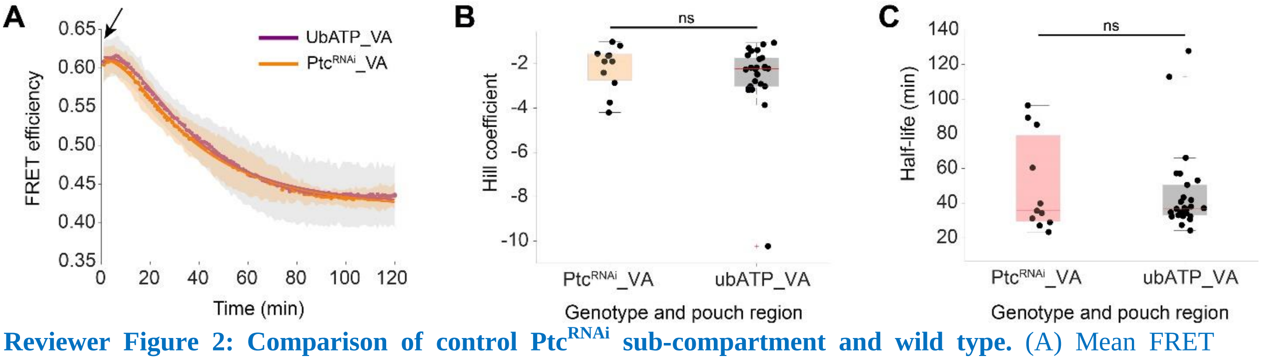

Reviewer Figure 2: Comparison of control Ptc^{RNAi} sub-compartment and wild type. (A) Mean FRET efficiency measured over time in the ventral anterior sub-compartment of wild type (UbATP_VA, Fig 1) or Ptc^{RNAi} (Fig 3C). Shaded regions indicate standard deviation (SD); dots indicate mean per timepoint, and solid lines indicate the fit to the mean. (B) Fit parameters of individual time traces for A. Each dot represents data from one disc. Box plots summarize the data (see Methods); ns = not significant p-value using a Kruskal-Wallis test (n=11 disc for Ptc^{RNAi} and n=26 discs for UbATP).

Minor comments

1. Line 82. hindered glycolysis upregulates Hh signaling - This was shown in the other paper. This should read something like "this work and previous..."

Response: In our rewrite of the introduction, this line was deleted. In the revised version, however, we have edited the text to be more explicit about what is shown in this work vs our previous study. For example, see lines 90-91, 271-73.

2. Section 1, Line 99. These areas are known growth "organizer" regions, with high signaling activity in the Wingless/Notch and Hedgehog pathways, respectively. - add a reference here

Response: We have now added references to two reviews covering patterning of the wing disc by these signaling centers (Tabata and Takei, 2004; Beira and Paro, 2016).

3. Extended data S1I - Why is the FRET efficiency after 60 min in S1I so different from the one in Fig1C?

Response: Please see the response to the Major comment #3 above. The figure in S1I is included to show that the region of slower ATP declines corresponds to the organizer regions at compartment boundaries. A more comprehensive analysis of the kinetics of ATP loss upon OxPhos inhibition is shown in Fig 1D-G. So as not to unnecessarily confuse the reader, we have now changed the images in Fig 1C to be closer to the mean and more similar to EV1J.

4. Extended data S1J - This image is not well explained in the main text.

Response: Thank you for pointing out this omission. We have added text to briefly explain it on lines 108-9.

5. Figure 1E-F and HI - Authors should state what is represented by the error bars and shaded regions.

Response: Thank you for pointing the lack of explanation in the figure legend. The shaded regions and error bars are actually box plots, with the boxes encompassing 2nd-3rd quartiles and the whisker lines denoting the 1st and 4th quartiles. We have revised the legend to indicate that these are box plots, and we have added an explanation of the box plots in the methods (lines 393-6).

6. Section 2, Line 139. What is the difference between these 2 inhibitors? Why combining it to block glycolysis? Add the rationale behind it to the text.

Response: We acknowledge that this point was not well-explained in the original manuscript. As metabolites can flow into the glycolysis pathway at different steps, we chose two inhibitors that act on different steps to be sure that we were sufficiently blocking glycolysis. We have added text on lines 141-4 to explain our choice.

7. Fig. 2C-D – Authors should state what is represented by the error bars.

Response: Changed as stated above.

8. Section 3, Fig S3B – ‘Shaded regions indicate SD; solid lines illustrate a fit of the mean data.’ This information should be found in S3C legend instead.

Response: Thank you for noticing this error. It has been corrected.

9. FigS3D-E – Authors should state what is represented by the error bars.

Response: Changed as stated above.

10. Fig 3E-F, H-I – Authors should state what is represented by the error bars.

Response: Changed as stated above.

11. Section 4, Fig4D-E – Authors should state what is represented by the error bars.

Response: Changed as stated above.

12. Authors talked about the insensitive control of the ATP biosensor and showed the range of FRET-values obtained with this tool. However, it would be good to have an image of a wing disc expressing ATP [RKRK] upon inhibition of glycolysis Oxphos? To confirm that no differences between OR and NO regions are observed.

Response: We assume that the reviewer is referring to the inhibition of OxPhos, which does show a pattern of metabolic activity with the ATP-sensitive sensor, rather than glycolysis, which does not. If so, the reviewer has correctly identified an important missing control. We have now added images and quantification (Fig EV1F-G) to show the pattern of FRET values from the ATP-insensitive RKRK construct upon OxPhos inhibition. As expected, there is no pattern or decrease in FRET efficiency values in the ATP-insensitive sensor upon antimycin addition, in contrast to the ATP-sensitive construct (Fig 1). We refer to this new data in the results text, lines 112-3.

13. Since authors suggested that glycolysis upregulation in the organizer region prevent a faster decline in ATP level, it would be interesting to see whether downregulation of glycolytic enzyme genes alters the pattern observed upon OxPhos inhibition (not required).

Response: We agree that this would be a nice experiment. Unfortunately, this experiment is complicated by the fact that we observe no pattern upon OxPhos inhibition in the control experiments of either *apGal*^{ts} or *nubGal*^{ts} drivers without any UAS construct (see above). Nevertheless, we did this experiment and found a very rapid decline in ATP levels upon OxPhos inhibition throughout the wing disc, indicating clearly that glycolytic enzymes are required for maintaining a certain level of ATP production during OxPhos inhibition. If requested, we can add this data (Reviewer Figure 3) to the paper, but have not included it in the revised version, as we do not feel that it strengthens the paper significantly.

14. Materials and Methods, Line 294. UAS-PtcRNAi (BDSC) - Authors should refer stock number

Response: After consulting our records, we realized that the origin of this line was actually unclear, and it did not match either of the two lines that currently exist in the BDSC, nor those in the VDRC. Thus, we sequenced the genome and identified it as matching a line in the NIG repository instead. A description is now provided in the methods (lines 482-510). Our sincere appreciation to the reviewer for drawing our attention to this important mistake!

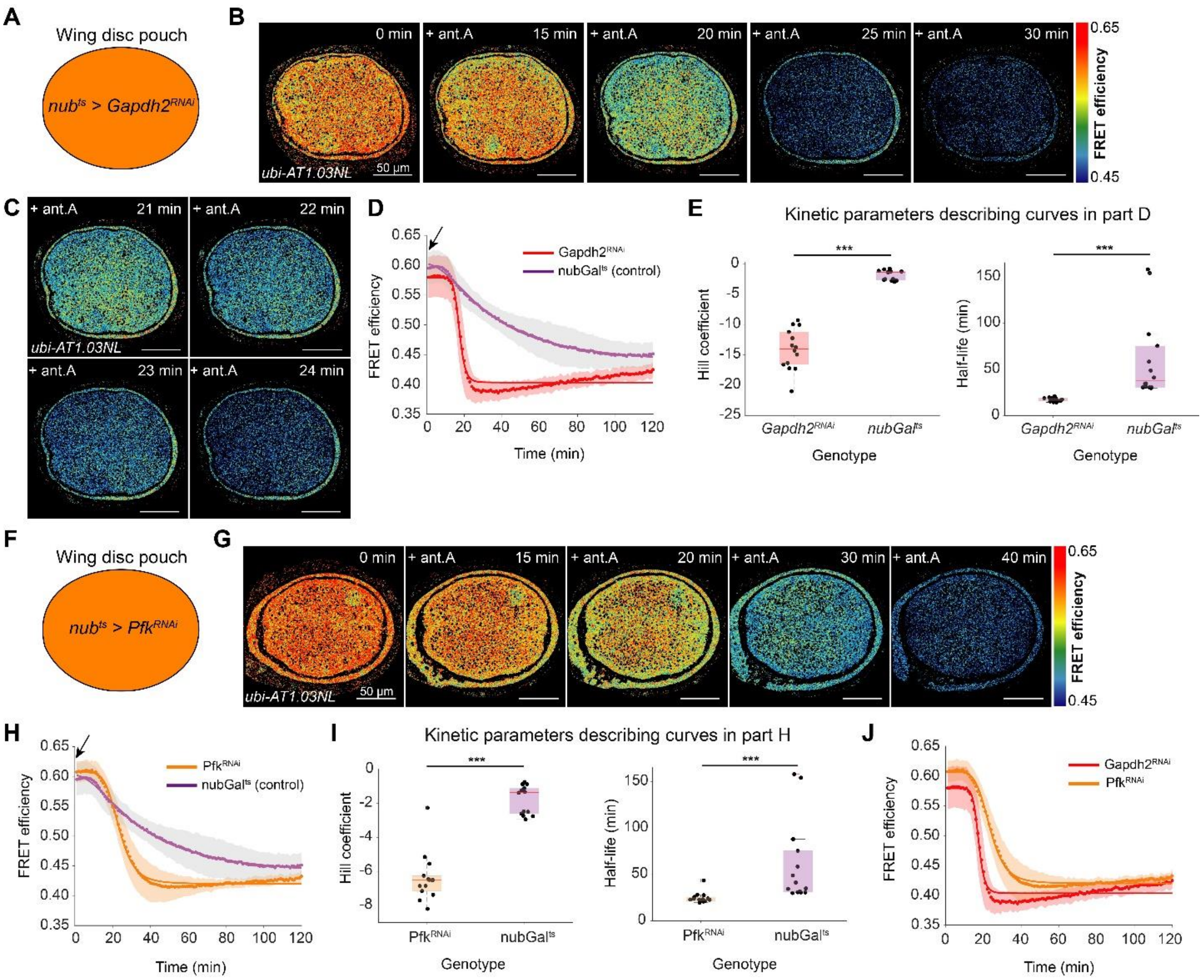

Reviewer Figure 3: Loss of glycolytic enzymes enhances kinetics of ATP decline upon OxPhos inhibition. (A, F) Schematics indicating the entire wing pouch, where the RNAi for Gapdh2 (A) and Phosphofructokinase (Pfk, F) was induced using *nubGal*^{ts}. (B, G) Timelapse montage of ATP sensor FRET efficiency in the *nubGal*^{ts} *>Gapdh2*^{RNAi} (B) or *nubGal*^{ts} *>Pfk*^{RNAi} (G) wing pouch after 10 µM antimycin A (ant.A) addition. (C) FRET efficiency of disc in (B) with increased temporal resolution (1 min intervals). (D, H) Mean FRET efficiency analyzed over time in *nubGal*^{ts} *>WT* (control discs, n = 14) and *nubGal*^{ts} *>Gapdh2*^{RNAi} discs (D, n = 14) or *nubGal*^{ts} *>Pfk*^{RNAi} discs (H, n = 14). Shaded regions indicate SD; dots represent the mean per timepoint; solid lines illustrate a fit of the mean data. (E, I) Kinetic parameters of the fit lines for ATP decline in *nubGal*^{ts} *>Gapdh2*^{RNAi} (E), *nubGal*^{ts} *>Pfk*^{RNAi} (I) and *nubGal*^{ts} *>WT* discs (E, I). Box plots summarize the data (see Methods). *** = p-value < 0.001 using a Kruskal-Wallis test. Black arrows indicate the addition of the drug.

Referee #2:

In this paper, Nellas et al explore the relationship between Hh signalling and ATP metabolism, using the Drosophila wing as a model. The Eaton group had previously shown with a FRET sensor that ATP levels modulate Hh signaling (Spannl, 2020). Here the authors report a reverse effect. Although ATP levels are relatively uniform in wing imaginal discs, relatively higher ATP is detected at the D/V and A/P boundaries upon inhibition of OxPhos. The focus of the present paper is on the residual ATP detected at the A/P boundary. From the result of double inhibition experiments, the authors suggest that residual ATP is caused by higher glycolysis. They then manipulate Hh signalling to test whether increased residual ATP at the A/P boundary is a consequence of increased Hh signalling there. They conclude that Hh signalling stimulates aerobic glycolysis, and that this could be part of a homeostatic mechanism since ATP levels have been suggested to boost Hh signalling activity.

Response: We thank the reviewer for the critical feedback on our work. Below, we provide a point-by-point response to the specific comments made.

1. The basic phenomenon described in this paper is somewhat contrived (a genetic effect is only seen in a situation of pharmacological inhibition). However, it can be argued that this is the way to uncover unsuspected activities.

Response: This is a valid point. As such, we designed an experiment to inhibit OxPhos without pharmacological perturbation. In Fig 4, we show that the same metabolic patterning occurs under oxygen deprivation, a much more natural way of inhibiting OxPhos and one that could be physiologically relevant during larval development. We have also considerably revised our introduction and added paragraphs to the end of the results section to better highlight the significance of this work and put it into a biological context.

2. Nevertheless, the significance of the observation remains limited because, even if Hh signalling boosts glycolysis, this must be a relatively minor phenomenon since higher ATP levels cannot be detected in untreated tissues. It is conceivable that any increase in glycolysis is compensated by a reduction in OxPhos (implying a different metabolic program at the organisers) but this is not considered by the authors.

Response: From this feedback, it became clear to us that we had not adequately explained our results and the logic of our experimental design. The altered text in the introduction and discussion (highlighted in blue throughout) should help address this point.

Importantly, it remains unclear whether the differences in organizers vs non-organizers are present always or only induced in response to OxPhos inhibition. We still think it is possible that the organizers in an unperturbed tissue are doing more glycolysis than the rest of the tissue, even though the overall steady state levels of ATP are not higher than elsewhere in the disc. It could indeed be, as the reviewer suggests, that this region produces less ATP from OxPhos. Alternatively, perhaps even more likely, the demand for ATP could be higher in this region, due to the energetic costs of signaling (endocytosis, phosphorylation cascades, etc). This idea is supported by the fact that we see a slightly higher rate of ATP decline in the organizers upon inhibiting both ATP production pathways, indicating that the consumption rate is higher (Fig 2B-C). Thus, this region may need to produce more ATP, perhaps even locally at the apical surface and rapidly to accommodate fluctuations. Ramping up glycolysis in the organizers could accommodate the increased demand in order to maintain the same global levels of ATP. It is impossible, however, to know the rates of ATP production from each pathway simply by reading the steady state concentrations of ATP. Assays to measure flux through these pathways could answer this question, but established assays for doing so are destructive and cannot easily reveal regional differences across the tissue. The FRET assay shown here, when combined with the pharmacological perturbation, can allow us to tease apart contributions of glycolysis and OxPhos to ATP production and measure rates of ATP consumption in a spatially resolved way. Furthermore, the nature of the assay allows us to assess changes on fast timescales, which is also very difficult, if not impossible, to measure in a developing tissue with genetic

perturbations alone. Thus, we argue that the methodology used in this paper is actually uniquely powerful and an advance in the field.

We hope this comment is largely addressed by the changes that we have made to the introduction and the later part of the combined results/discussion sections, where we now discuss these ideas, including the possibility that there is increased energetic demand at the organizers due to signaling and the specific role that glycolysis may play in supplying energy.

3. The authors conclude from the double inhibition experiments that glycolysis is higher at the organisers than elsewhere. However, one cannot exclude the possibility that these cells could be particularly resistant to Oxphos inhibition or that inhibition of Oxphos leads to differential changes in ATP turnover.

Response: The reviewer raises a valid point that we did not consider that organizer cells may be particularly resistant to OxPhos inhibition. With only the antimycin A data in our original manuscript, this is a reasonable possibility. However, we now have data from oxygen depletion (Fig 4), which agrees very well with our results using antimycin A (Fig 1). For the reviewer's information, we also observed the same effect with the addition of oligomycin, a different inhibitor of OxPhos, making it unlikely that our results are due to any potential variations in drug permeabilization (data can be added upon request). Thus, we have now used multiple methods for inhibiting OxPhos and obtain the same results. If it were true that the slower kinetics of ATP decline at the organizer regions were due to some kind of general resistance to OxPhos inhibition of any sort, this effect should be unchanged by inhibition of glycolysis. The fact that the combined inhibition of glycolysis and OxPhos makes organizers behave more similar to the rest of the disc strongly suggests that the organizers have increased glycolysis.

As for the second point, that there may be differential changes in ATP turnover upon OxPhos inhibition. It is indeed our assumption that OxPhos inhibition itself does not change the consumption rate differentially across the tissue. It is a formal possibility that during OxPhos inhibition, the organizers slow down consumption more than the rest of the tissue. If this were occurring, however, additional inhibition of glycolysis would likely not have any effect. Double inhibition of OxPhos and glycolysis actually reveals a slightly faster ATP depletion in the organizers (Fig 2C), indicating that they consume ATP faster than the rest. Thus, we think the most reasonable explanation is that the organizers show slower ATP decline upon OxPhos inhibition because of differences in production (more glycolysis) rather than consumption.

In the revised version, we added more explanation to make the logic underlying this experiment more transparent to the reader, in particular in the results section, lines 126-40.

4. I do not understand the effect of CiDN in the P compartment. There is no Hh signaling in this compartment so it is not clear how further inhibiting Hh signalling would have an effect, unless, as the authors imply, this is due to a neomorphic activity of CiDN. In this case, an alternative means of inhibiting Hh signalling (smo RNAi?) is needed.

Response: Our results suggest that CiDN represses genes that are required for glycolysis. We think these are likely to be normally regulated by Ci in the anterior compartment, but in the posterior compartment they must be regulated by something else since Ci is not there. Unfortunately, we are limited to gain-of-function perturbations with the Gal4/UAS system. As stated above in the response to Reviewer 1, the *apGal*^{ts} (as well as *nubGal*^{ts}) itself alters the pattern of ATP decline upon OxPhos inhibition. Thus, it would be very difficult to detect differences caused by loss-of-function perturbations driven by Gal4, including Smo^{RNAi}. Nonetheless, the CiDN allele is an established way of perturbing the Hh pathway and our results are consistent with our claim that Hh positively regulates glycolysis upon OxPhos inhibition.

5. It is not clear what the authors mean by a homeostatic mechanism as a way to link the present study to the previous publication of this group.

Response: We thank the reviewer for pointing out our inadequate explanation. We have considerably revised the introduction and the last part of the results/discussion section to explain our ideas, which also better highlights the wider significance of these results. Specifically, we include a discussion of the homeostatic loop on lines 265-77.

6. As it stands this manuscript reports an interesting observation but fails to properly document the underlying mechanism or its wider significance.

Response: We have taken this comment seriously and have significantly revised the text to better highlight the wider significance of our work. Determining the molecular mechanism would be beyond the scope of this short report, however the addition of the oxygen deprivation experiment (Fig 4) elevates the paper by providing potential physiological relevance.

7. Additional comment: In fig. 3C, the Ptc RNAi effect seems to spread into P compartment. Is this real?

Response: We compared the posterior compartments on the dorsal (Ptc^{RNAi}-expressing) and ventral (no Ptc^{RNAi}) sides and found no statistical difference (Fig EV4C-D). Thus, we do not find solid evidence for a spreading into the P compartment, as the reviewer suggests. We acknowledge that it was difficult for the reader to discern the locations of the AP boundary in the original figure Fig 3C. To alleviate this problem, we have now added a red dashed line to this figure indicating the location of AP boundary. With this addition, it is also visibly clearer that there is not much effect on the posterior compartment.

Referee #3:

In this manuscript, Nellas, et al follow up on recent work from the lab (Spanni, et al) to assess the impact of altering glucose metabolism mechanisms on ATP generation in the Drosophila wing disc and define how the Hedgehog signaling pathway contributes to the process. Using a FRET sensor for ATP, the authors report differential impact of oxidative phosphorylation inhibition in "organizer" regions at the anterior-posterior (AP) and dorsal-ventral (DV) boundaries of the wing disc. Slower decay of FRET is observed in the organizer regions, suggesting that ATP is retained for longer than in "non-organizer" cells. Doubly inhibiting oxidative phosphorylation and glycolysis eliminates the delayed response, supporting a model in which glycolysis remains active in organizer regions for longer after inhibition of oxidative phosphorylation. Extending recently published work, the authors demonstrate increased FRET upon constitutive activation of Hedgehog signaling (ptc knockdown) and accelerated FRET loss upon overexpression of a dominant negative form of Ci. Strengths of the work include quantitative measurements of ATP levels in real time and space in a developing tissue, a technically challenging, but critically important problem. The question of the developmental roles and signaling impacts of carbohydrate metabolism is also of high importance, with this work addressing how inhibition of the major pathway (OxPhos) impacts the minor (glycolysis). The study complements the lab's recently published work that identified glycolysis as a regulator of Hh signaling amplitude via promoting Smo retention at the plasma membrane. Here, the reverse process is assessed, with measurement of the impact of Hh signaling on ATP generation via glycolysis. The two papers together support the existence of a negative feedback loop, whereby Hh signaling activates glycolysis and then glycolysis limits Hh signaling through regulation of plasma membrane potential and entry of lipid-based Hh pathway inhibitors. Here, the impact of Hh signaling on ATP production is clearly demonstrated and the links to glycolysis are reasonably well supported using drug inhibition studies.

Response: We thank the reviewer for the positive feedback on our work. Below, we provide a point-by-point response to address the specific issues raised.

1. The manuscript could benefit from addition of a biologically relevant context for the reported studies. For example, there are a number of ways that oxidative phosphorylation might be inhibited during larval development. Discussion of how Hh-mediated induction of glycolysis in organizer cells upon natural depletion of ATP via OxPhos inhibition (oxygen deprivation, starvation, etc) might impact wing disc cells and tissue and what the implications might if the mechanism is conserved in other Hh-dependent cells would provide a stronger justification for the relevance of the study for understanding the biology. This may require tightening up of the Introduction and Results to remain within the character limits, but would really provide a strong framework for the study and its implications.

Response: The reviewer makes an important point and we have taken this comment seriously. In the revised version, we have added new data showing the metabolic response to oxygen deprivation (new Fig 4), a more natural inhibition of OxPhos that could be physiologically relevant. Strikingly, our results are very similar to our previous data on OxPhos inhibition with antimycin A (Fig 1): organizer regions lose ATP with slower kinetics than the rest of the wing disc. This result indicates that signaling centers may be preferentially spared during brief episodes of hypoxia. We incorporate these new data into a more elaborate discussion, which is now present at the end of the results section in the revised manuscript. We have also significantly reworked the introduction to better introduce the question and place this work into a broader context, while staying within the character limits of a report.

With regard to what implications there may be for other Hh-dependent cells, we have already highlighted in the introduction two other studies showing that Hh can promote aerobic glycolysis, indicating that it does seem to be broadly conserved (Teperino et al., 2012; Gershon et al., 2013; Di Magno et al., 2014). Without further information on the underlying mechanism (which is beyond the scope of this report), however, it is premature to speculate about how it may be relevant for other Hh-dependent phenomena, particularly in cancer or vertebrate development. Nonetheless, the changes that

we have made to the introduction and discussion should be sufficient to emphasize the significance of this work for understanding tissue development and the connection between morphogen signaling and energy homeostasis.

2. The nature of FRET experiments is that they are variable in living tissues, and sensitivity can be a challenge. As such, the differences between conditions are small throughout the manuscript. In addition, glycolysis appears to be more of a back-up plan for the tissue than the primary energy production machine, adding to the relatively small effects. Placement of the work into a context of how this might be relevant in vivo, as suggested above, would increase the impact of the findings. Of note, the extensive statistical analysis largely mitigates the magnitude issue.

Response: We appreciate the reviewer's acknowledgement of the problem and recognition of our statistical analysis. This comment made us realize that we did not adequately explain our data and why small effects could still be relevant. As stated above, we have now modified the introduction and added new paragraphs at the conclusion of the results section to address this point. In short, while the contribution of glycolysis to energy production may be small, it could still be important by preferentially supporting particular energy-demanding processes, such as maintenance of the plasma membrane potential, which require fast and potentially localized energy production. Morphogen signaling may further increase energetic burden at organizer regions and thereby introduce more energy stress that can be quickly accommodated by glycolysis. It is a strength of the ATP FRET sensor, which enables precise spatial-temporal monitoring of ATP dynamics, that we can detect such subtilties in energy metabolism. More traditional methods, which are often destructive, require longer timescales, and are not spatially resolved, are unable to catch some of this behavior, precisely because energy metabolism is dynamic and robust.

3. It would be helpful to have figures labeled more clearly with genotype and drug status. For example, it takes jumping back and forth from Figure 3 to the text to determine that 3E is untreated, ptc KD and 3F is antimycin A-treated, ptc KD. This is an issue throughout the figures. Labeling the figure itself or making the legend more clear would be helpful.

Response: We apologize for the lack of clarity and have made efforts to make the figures more readable, including adding titles to the figure subsections and enhancing readability of the genotype labels in the timelapse montage images.

Dear Dr. Dye,

Thank you for submitting your revised manuscript. It has now been seen by all of the original referees.

As you can see, the referees find that the study is significantly improved during revision and recommend publication. However, I need you to address the editorial points below before I can accept the manuscript.

• Please address the remaining concerns of referee #2 either experimentally, or by textually discussing the points in the discussion section. Please provide a point-by-point response as well.
• We updated our journal's competing interests policy in January 2022 and request authors to consider both actual and perceived competing interests. Please review the policy https://www.embopress.org/competing-interests and update your competing interests if necessary. Also, please rename the 'Conflict of Interests' section as 'Disclosure statement and competing interests'.
• As per our format requirements, in the reference list, citations should be listed in alphabetical order and then chronologically, with the authors' surnames and initials inverted; where there are more than 10 authors on a paper, 10 will be listed, followed by 'et al.'. Please see https://www.embopress.org/page/journal/14693178/authorguide#referencesformat
• We note that Fig 3J&K, EV3A-D, EV5A-I and Appendix Fig S1A-C,E&G are currently not called out in the text.
• Please add a Table of Contents to the Appendix file. Moreover, the nomenclature should be Appendix Figure S1.
• Papers published in EMBO Reports include a 'synopsis' and 'bullet points' to further enhance discoverability. Both are displayed on the html version of the paper and are freely accessible to all readers. The synopsis includes a short standfirst summarizing the study in 1 or 2 sentences (max 35 words) that summarize the paper and are provided by the authors and streamlined by the handling editor. I would therefore ask you to include your synopsis blurb and 3-5 bullet points listing the key experimental findings.
• In addition, please provide an image for the synopsis. This image should provide a rapid overview of the question addressed in the study but still needs to be kept fairly modest since the image size cannot exceed 550x400 pixels.
• Our production/data editors have asked you to clarify several points in the figure legends (see attached document). Please incorporate these changes in the attached word document and return it with track changes activated.

Thank you again for giving us to consider your manuscript for EMBO Reports, I look forward to your minor revision.

Kind regards,

Deniz Senyilmaz Tiebe

--
Deniz Senyilmaz Tiebe, PhD
Editor
EMBO Reports

Referee #1:

The authors addressed all the major and minor comments. When needed, authors prepared additional experiments to address the questions raised.
The manuscript is now clearer about the major findings and relevance of the work. These alterations significantly improved the quality of the manuscript.
I recommend the publication of this work.

Referee #2:

The authors have made an effort to clarify various aspects of the manuscript, but uncertainty continues to linger. Sensitivity of the phenomenon to genetic background casts doubt about its generality and the role of Hh signalling remains incompletely documented.
The value of publishing these findings is that they can be scrutinised by others but, as they stand the conclusions of the present study should be taken as preliminary.

A few specific comments:

As pointed out by reviewer 1, the control compartment in the experiment with apGalts does not behave like wildtype (no boundary effect). The authors suggest that this could be due to the presence of Gal. This seems unlikely since, in this instance, Gal4 is not expressed in the control compartment. There is a concern therefore that the phenomenon uncovered by the authors

may not be reproducible in a variety of genetic background, casting doubt on the generality of their conclusions.

In response to reviewer 2, the authors suggest that organiser cells consume and produce more ATP, as this would explain the seemingly uniform ATP levels in undisturbed discs. They may be right but, without a direct measure of turnover rate, this remains un unproven hypothesis. As an aside, I find the authors' rationalisation that organiser cells are more energy-hungry than non-organiser cells unconvincing.

The value of the experiment with CiDN remains unclear. The authors acknowledge that CiDN functions independently of Hh signalling in the P compartment. How can they exclude the possibility that this could also be the case in the A compartment? Without an alternative means of preventing Hh transduction, a direct role of Hh signalling remains unproven. It is also not clear why Gal4 can be used to express CiDN (as it is in this particular experiment) but not for other UAS-transgene.

Referee #3:

Nellas, et al have uncovered an interesting phenomenon whereby ATP production differentially depends on glycolysis versus oxidative phosphorylation in different regions of the developing wing. Measurements of ATP in a developing organism using a FRET-based reporter are combined with use of drugs to inhibit OxPhos, glycolysis or both to identify the regional and molecular regulation of this important metabolic process.

As in the initial submission, the technical aspects of the manuscript are robust, with statistically significant differences observed. Now, however, the authors really uncover the biological relevance of their observations, with a quite remarkable experiment showing that oxygen deprivation in living larvae under the microscope causes the same effects! My initial review merely requested written clarification of the context of the work, but the authors went ahead and demonstrated in vivo relevance of the mechanism experimentally, providing an exciting framework for metabolic switching in a biological context.

The paper is very well written, provides an interesting and novel advance to the field, and leverages ex vivo imaging and challenging in vivo approaches to address how metabolic compensation can assure normal development to proceed even under conditions where one mechanism is inhibited. With the new data demonstrating that this mechanism is observed under conditions that would normally be experienced by larvae, the developmental context is very interesting, with potential broad implications for metabolic switching as an insurance mechanism for normal development to proceed under stress. Identification of the Hh pathway as an important regulator of this process adds to the impact of the work. Conservation of this mechanism also may have important implications for Hh-dependent cancers, which rely on glycolysis and OxPhos for energy production, depending on the environmental context. For these reasons, I recommend publication in EMBO Reports.

Nellas et al: Response to Reviewer comments

Thanks again to the reviewers for their further evaluation of our work. We have again revised the manuscript in order to address remaining uncertainties.

To summarize the changes present in the new revised version:

- We added data from a new experiment (Reviewer Fig 4), where we perturb the Hh pathway with a GAL4-independent method, using a temperature-sensitive allele of Hh. The data from this experiment address comments 1 and 3 of Reviewer 2 and strengths the data underlying the conclusions of this manuscript.
- Slight adjustments to the text were made on line 265 to address comment 2 of Reviewer 2, as well as on line 241 to respond to a comment made by Reviewer 3.
- Changes to formatting and clarifications of the text requested by the editorial staff have all been made (e.g., references, figure legends, figure citations in the text – please note the tracked changes).

Reviewer 1:

The authors addressed all the major and minor comments. When needed, authors prepared additional experiments to address the questions raised. The manuscript is now clearer about the major findings and relevance of the work. These alterations significantly improved the quality of the manuscript. I recommend the publication of this work.

Thank you for the positive feedback on our work.

Referee #2:

The authors have made an effort to clarify various aspects of the manuscript, but uncertainty continues to linger. Sensitivity of the phenomenon to genetic background casts doubt about its generality and the role of Hh signalling remains incompletely documented. The value of publishing these findings is that they can be scrutinised by others but, as they stand the conclusions of the present study should be taken as preliminary.

A few specific comments:

1. As pointed out by reviewer 1, the control compartment in the experiment with apGalts does not behave like wildtype (no boundary effect). The authors suggest that this could be due to the presence of Gal. This seems unlikely since, in this instance, Gal4 is not expressed in the control compartment. There is a concern therefore that the **phenomenon uncovered by the authors may not be reproducible in a variety of genetic background,** casting doubt on the generality of their conclusions.

We provide two additional pieces of information that indicate our results are not confounded by other mutations in the background of the UbATP fly line:

1. Outcrossing the UbATP flies to wild type (OreR), which should reduce the effect of genetic background, does not change our results: we still observe a patterned response to OxPhos inhibition, where ATP declines slower at the organizers than elsewhere in the tissue. Data for this experiment can be provided upon request.

2. We have now recombined the UbATP sensor with HhTS mutant on the same chromosome and then crossed this line with another Hh mutant fly line, Hh[AC]. This genetic background should be significantly different from our original line. Under permissive conditions for Hh signaling (18C), we still see slower ATP decline at the AP boundary upon OxPhos inhibition. These data are shown below in Reviewer Figure 4A.

Thus, the result that the organizers behave differently to OxPhos inhibition than the rest of the wing pouch is robust in fly lines that do not express GAL4, alleviating the concern that the effect is not robust to genetic background.

2. In response to reviewer 2, the authors suggest that organiser cells consume and produce more ATP, as this would explain the seemingly uniform ATP levels in undisturbed discs. They may be right but, without a direct measure of turnover rate, this remains un unproven hypothesis. As an aside, I find the authors' rationalisation that organiser cells are more energy-hungry than non-organiser cells unconvincing.

We do directly measure consumption rate in different regions of the wing pouch by inhibiting both ATP production pathways (glycolysis and OxPhos) and measuring the decline of ATP concentrations with our FRET-ATP reporter (Fig 2A-C). We do not know of an alternative method for measuring ATP turnover that could address this comment. Our method uniquely allows us to measure ATP turnover in an intact tissue in real time, in a spatially resolved manner.

The suggestion that the reviewer objects to, "the authors suggest that organiser cells consume and produce more ATP, as this would explain the seemingly uniform ATP levels in undisturbed discs", was made specifically to answer the reviewer's question in the last round, and it appears only in the written response to the reviewers, not in the manuscript.

We agree that we can only hypothesize about the completely unperturbed state – our conclusions, however, come from our observations about what happens upon inhibiting ATP producing pathways. In the text of the results section of the manuscript, we always qualify our observations by saying that we are observing what happens **in response to inhibition of OxPhos**. For example, "Our data from Fig 1 indicate that although steady state levels of ATP are spatially uniform, differences in metabolism between organizers and non-organizers are revealed when ATP production from OxPhos is blocked." As another example, "This finding indicates that **upon OxPhos inhibition**, glycolysis still generates ATP in the entire wing pouch but more so in the organizer regions." It should always be clear to the reader that we made conclusions about how the cells **respond to** OxPhos inhibition. The only general statement we make about the completely unperturbed state comes late, during the discussion: "Importantly, future work will be needed to determine whether the observed differences in ATP metabolism between organizer and non-organizer regions are only induced in response to OxPhos inhibition, or whether they are already present prior to the perturbation." The rest of the sentences in this paragraph (lines 258-68) were added in order to provide some further interpretation and broader context, as was requested in the last round of revisions. We have now further adjusted this section – shortening our hypotheses about increased energetic burden at signaling centers and adding the language "could suggest" and "We propose that…" in order to make it very clear to the reader that we are making a hypothesis and not a conclusion.

Importantly, even if the phenomenon we describe in this paper – elevated ATP consumption and glycolytic ATP production at organizers – turns out to be only relevant during OxPhos inhibition, it is still an interesting, important, and potentially physiologically relevant observation, as it indicates that signaling centers of the wing pouch are energetically more plastic and potentially spared during energy deprivation (as exemplified by the hypoxia experiment).

3. The value of the experiment with CiDN remains unclear. The authors acknowledge that CiDN functions independently of Hh signalling in the P compartment. How can they exclude the possibility

that this could also be the case in the A compartment? Without **an alternative means of preventing Hh transduction,** a direct role of Hh signalling remains unproven. It is also not clear why Gal4 can be used to express CiDN (as it is in this particular experiment) but not for other UAS-transgene.

The reviewer raises important points, which we have now addressed with a new experiment shown in Reviewer Figure 4. Here, we perturb the pathway using a GAL4-independent method, using a well-established temperature-sensitive allele of Hh (Ma et al Cell 1993). As described above in the response to the first comment, we recombined the UbATP FRET sensor with a Hh temperature-sensitive mutant allele and then crossed it with a separate Hh loss-of-function allele, Hh[AC] (Lee et al Cell 1992). This genetic combination (HhTS/Hh[AC]) has been used previously in the literature to reduce Hh signaling upon temperature shift (e.g., Chang et al PNAS 2013). At the permissive temperature (18C), we observe a patterned response to OxPhos inhibition that is similar to that of wild type at 25C: ATP declines slower in organizer regions (Reviewer Figure 4A). After a 24hr shift to restrictive temperature, however, the patterned response is no longer visible: the blade behaves similarly throughout the wing pouch (Reviewer Figure 4B). The wing pouch is also smaller when grown at restrictive temperatures, consistent with the loss of Hh pathway. The kinetics of ATP depletion are clearly faster at 30C (compare part B with part A), likely because of the higher temperature. To verify that the shift in temperature alone cannot destroy the patterned response to OxPhos inhibition, we shifted wild type flies to 30C and find that the organizers still lose ATP slower than elsewhere in the pouch (Reviewer Figure 4C). Thus, the loss of the pattern in the HhTS/Hh[AC] discs (Reviewer Figure 4B) is due to the loss of Hh activity.

These data are consistent with the CiDN experiment and support our statement that the Hh pathway is required for the upregulation of glycolytic ATP at the AP boundary upon OxPhos inhibition. We would like to keep the CiDN experiment in the main paper because the approach parallels the PtcRNAi experiment. Given the length restrictions for a report, we also prefer to keep this figure in the response to reviewers.

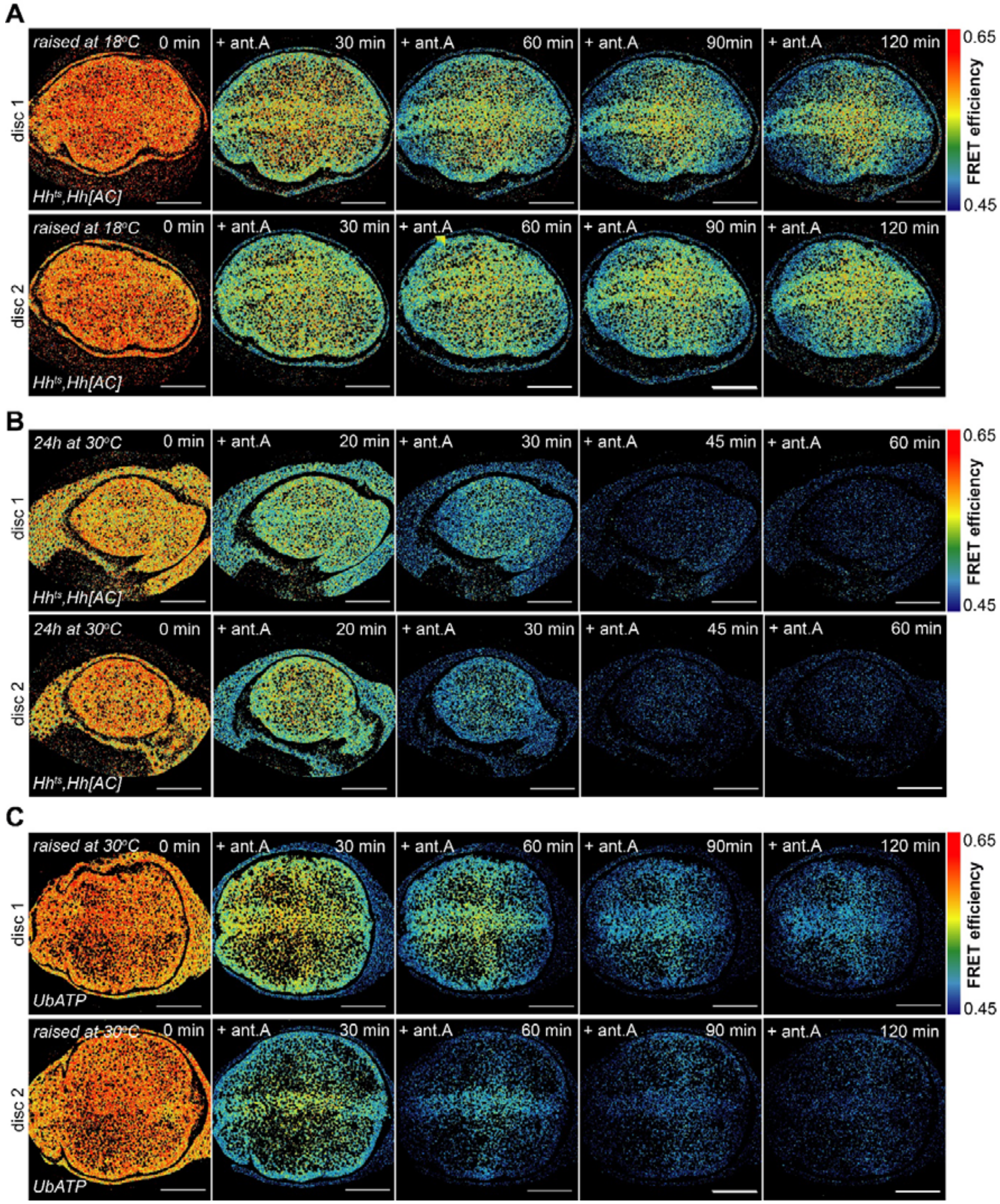

Reviewer Figure 4. Loss of Hh protein activity reduces patterned glycolytic ATP production. Timelapse montages of ATP sensor FRET efficiency in wing disc explants of different genotypes and conditions upon OxPhos inhibition. (A) Timelapse montages of ATP sensor FRET efficiency in wing disc explants from Hhts/Hh[AC] larvae raised at 18C phenocopy the patterned response to OxPhos inhibition (Fig1). (B) Timelapse montages of ATP sensor FRET efficiency in wing disc explants from Hhts/Hh [AC] larvae shifted to 30C (loss of Hh activity) do not show a patterned response to OxPhos inhibition (representatives of 13 discs out of 3 independent experiments). (C) Timelapse montages of ATP sensor FRET efficiency in wing disc explants from UbATP larvae raised at 30C demonstrate that the patterned response to OxPhos inhibition persists after shift to 30C.

Referee #3:

Nellas, et al have uncovered an interesting phenomenon whereby ATP production differentially depends on glycolysis versus oxidative phosphorylation in different regions of the developing wing. Measurements of ATP in a developing organism using a FRET-based reporter are combined with use of drugs to inhibit OxPhos, glycolysis or both to identify the regional and molecular regulation of this important metabolic process.

As in the initial submission, the technical aspects of the manuscript are robust, with statistically significant differences observed. Now, however, the authors really uncover the biological relevance of their observations, with a quite remarkable experiment showing that oxygen deprivation in living larvae under the microscope causes the same effects! My initial review merely requested written clarification of the context of the work, but the authors went ahead and demonstrated in vivo relevance of the mechanism experimentally, providing an exciting framework for metabolic switching in a biological context.

The paper is very well written, provides an interesting and novel advance to the field, and leverages ex vivo imaging and challenging in vivo approaches to address how metabolic compensation can assure normal development to proceed even under conditions where one mechanism is inhibited. With the new data demonstrating that this mechanism is observed under conditions that would normally be experienced by larvae, the developmental context is very interesting, with potential broad implications for metabolic switching as an insurance mechanism for normal development to proceed under stress. Identification of the Hh pathway as an important regulator of this process adds to the impact of the work. Conservation of this mechanism also may have important implications for Hh-dependent cancers, which rely on glycolysis and OxPhos for energy production, depending on the environmental context. For these reasons, I recommend publication in EMBO Reports.

Thank you for the positive feedback on our work. To be clear, however, we performed the hypoxia experiment in explants, not in intact living larvae. To prevent this confusion in future readings, we added the phrase "in explants" to line 241 where we first introduce this experiment, as well as to the figure legend for Fig 4A.

2nd Revision - Editorial Decision 22nd Aug 2022

Dr. Natalie Dye
MPI of Molecular Cell Biology & Genetics
Pfotenhauerstr. 108
D-01307 Dresden
Germany

Dear Natalie,

Thank you for incorporating the former reviewer figure into the Appendix. I have uploaded the files you sent and am now very pleased to accept your manuscript for publication in the next available issue of EMBO reports. Thank you for your contribution to our journal.

At the end of this email I include important information about how to proceed. Please ensure that you take the time to read the information and complete and return the necessary forms to allow us to publish your manuscript as quickly as possible.

As part of the EMBO publication's Transparent Editorial Process, EMBO reports publishes online a Review Process File to accompany accepted manuscripts. As you are aware, this File will be published in conjunction with your paper and will include the referee reports, your point-by-point response and all pertinent correspondence relating to the manuscript.

If you do NOT want this File to be published, please inform the editorial office within 2 days, if you have not done so already, otherwise the File will be published by default [contact: emboreports@embo.org]. If you do opt out, the Review Process File link will point to the following statement: "No Review Process File is available with this article, as the authors have chosen not to make the review process public in this case."

Thank you again for your contribution to EMBO reports and congratulations on a successful publication. Please consider us again in the future for your most exciting work.

Kind regards,

Martina

Martina Rembold, PhD
Senior Editor
EMBO reports

****

THINGS TO DO NOW:

Please note that you will be contacted by Wiley Author Services to complete licensing and payment information. The required 'Page Charges Authorization Form' is available here: https://www.embopress.org/pb-assets/embo-site/er_apc.pdf - please download and complete the form and return to embopressproduction@wiley.com

You will receive proofs by e-mail approximately 2-3 weeks after all relevant files have been sent to our Production Office; you should return your corrections within 2 days of receiving the proofs.

Please inform us if there is likely to be any difficulty in reaching you at the above address at that time. Failure to meet our deadlines may result in a delay of publication, or publication without your corrections.

All further communications concerning your paper should quote reference number EMBOR-2021-54025V3 and be addressed to emboreports@wiley.com.

Should you be planning a Press Release on your article, please get in contact with emboreports@wiley.com as early as possible, in order to coordinate publication and release dates.

EMBO Press Author Checklist

Corresponding Author Name: Natalie Anne Dye	
Journal Submitted to: EMBO Reports	
Manuscript Number: EMBOR-2021-54025V1	

USEFUL LINKS FOR COMPLETING THIS FORM
The EMBO Journal - Author Guidelines
EMBO Reports - Author Guidelines
Molecular Systems Biology - Author Guidelines
EMBO Molecular Medicine - Author Guidelines

Reporting Checklist for Life Science Articles (updated January 2022)
This checklist is adapted from Materials Design Analysis Reporting (MDAR) Checklist for Authors. MDAR establishes a minimum set of requirements in transparent reporting in the life sciences (see Statement of Task: 10.31222/osf.io/9sm4x). Please follow the journal's guidelines in preparing your manuscript.
Please note that a copy of this checklist will be published alongside your article.

Abridged guidelines for figures
1. Data
The data shown in figures should satisfy the following conditions:
- the data were obtained and processed according to the field's best practice and are presented to reflect the results of the experiments in an accurate and unbiased manner.
- ideally, figure panels should include only measurements that are directly comparable to each other and obtained with the same assay.
- plots include clearly labeled error bars for independent experiments and sample sizes. Unless justified, error bars should not be shown for technical replicates.
- if n<5, the individual data points from each experiment should be plotted. Any statistical test employed should be justified.
- Source Data should be included to report the data underlying figures according to the guidelines set out in the authorship guidelines on Data Presentation.

2. Captions
Each figure caption should contain the following information, for each panel where they are relevant:
- a specification of the experimental system investigated (eg cell line, species name).
- the assay(s) and method(s) used to carry out the reported observations and measurements.
- an explicit mention of the biological and chemical entity(ies) that are being measured.
- an explicit mention of the biological and chemical entity(ies) that are altered/varied/perturbed in a controlled manner.
- the exact sample size (n) for each experimental group/condition, given as a number, not a range;
- a description of the sample collection allowing the reader to understand whether the samples represent technical or biological replicates (including how many animals, litters, cultures, etc.).
- a statement of how many times the experiment shown was independently replicated in the laboratory.
- definitions of statistical methods and measures:
  - common tests, such as t-test (please specify whether paired vs. unpaired), simple χ2 tests, Wilcoxon and Mann-Whitney tests, can be unambiguously identified by name only, but more complex techniques should be described in the methods section;
  - are tests one-sided or two-sided?
  - are there adjustments for multiple comparisons?
  - exact statistical test results, e.g., P values = x but not P values < x;
  - definition of 'center values' as median or average;
  - definition of error bars as s.d. or s.e.m.

Please complete ALL of the questions below.
Select "Not Applicable" only when the requested information is not relevant for your study.

Materials

Newly Created Materials	Information included in the manuscript?	In which section is the information available? (Reagents and Tools Table, Materials and Methods, Figures, Data Availability Section)
New materials and reagents need to be available; do any restrictions apply?	Not Applicable	

Antibodies	Information included in the manuscript?	In which section is the information available? (Reagents and Tools Table, Materials and Methods, Figures, Data Availability Section)
For **antibodies** provide the following information:<br>- Commercial antibodies: RRID (if possible) or supplier name, catalogue number and or/clone number<br>- Non-commercial: RRID or citation	Yes	Materials and Methods section

DNA and RNA sequences	Information included in the manuscript?	In which section is the information available? (Reagents and Tools Table, Materials and Methods, Figures, Data Availability Section)
**Short novel DNA or RNA including primers, probes**: provide the sequences.	Not Applicable	

Cell materials	Information included in the manuscript?	In which section is the information available? (Reagents and Tools Table, Materials and Methods, Figures, Data Availability Section)
**Cell lines:** Provide species information, strain. Provide accession number in repository **OR** supplier name, catalog number, clone number, and/**OR** RRID.	Not Applicable	
**Primary cultures:** Provide species, strain, sex of origin, genetic modification status.	Not Applicable	
Report if the cell lines were recently **authenticated** (e.g., by STR profiling) and tested for mycoplasma contamination.	Not Applicable	

Experimental animals	Information included in the manuscript?	In which section is the information available? (Reagents and Tools Table, Materials and Methods, Figures, Data Availability Section)
**Laboratory animals or Model organisms:** Provide species, strain, sex, age, genetic modification status. Provide accession number in repository **OR** supplier name, catalog number, clone number, **OR** RRID.	Yes	Materials and Methods
**Animal observed in or captured from the field:** Provide species, sex, and age where possible.	Not Applicable	
Please detail **housing and husbandry conditions**.	Yes	Materials and Methods

Plants and microbes	Information included in the manuscript?	In which section is the information available? (Reagents and Tools Table, Materials and Methods, Figures, Data Availability Section)
**Plants:** provide species and strain, ecotype and cultivar where relevant, unique accession number if available, and source (including location for collected wild specimens).	Not Applicable	
**Microbes:** provide species and strain, unique accession number if available, and source.	Not Applicable	

Human research participants	Information included in the manuscript?	In which section is the information available? (Reagents and Tools Table, Materials and Methods, Figures, Data Availability Section)
If collected and within the bounds of privacy constraints report on age, sex and gender or ethnicity for all study participants.	Not Applicable	

Core facilities	Information included in the manuscript?	In which section is the information available? (Reagents and Tools Table, Materials and Methods, Figures, Data Availability Section)
If your work benefited from core facilities, was their service mentioned in the acknowledgments section?	Yes	Acknowledgements

Design

Study protocol	Information included in the manuscript?	In which section is the information available? (Reagents and Tools Table, Materials and Methods, Figures, Data Availability Section)
If study protocol has been **pre-registered, provide DOI in the manuscript**. For clinical trials, provide the trial registration number **OR** cite DOI.	Not Applicable	
Report the **clinical trial registration number** (at ClinicalTrials.gov or equivalent), where applicable.	Not Applicable	

Laboratory protocol	Information included in the manuscript?	In which section is the information available? (Reagents and Tools Table, Materials and Methods, Figures, Data Availability Section)
Provide DOI OR other citation details if **external detailed step-by-step protocols** are available.	Not Applicable	

Experimental study design and statistics	Information included in the manuscript?	In which section is the information available? (Reagents and Tools Table, Materials and Methods, Figures, Data Availability Section)
Include a statement about **sample size** estimate even if no statistical methods were used.	Not Applicable	
Were any steps taken to minimize the effects of subjective bias when allocating animals/samples to treatment (e.g. **randomization procedure**)? If yes, have they been described?	Not Applicable	
Include a statement about **blinding** even if no blinding was done.	Not Applicable	
Describe **inclusion/exclusion criteria** if samples or animals were excluded from the analysis. Were the criteria pre-established? If sample or data points were omitted from analysis, report if this was due to attrition or intentional exclusion and provide justification.	Yes	Materials and Methods
For every figure, are **statistical tests** justified as appropriate? Do the data meet the assumptions of the tests (e.g., normal distribution)? Describe any methods used to assess it. Is there an estimate of variation within each group of data? Is the variance similar between the groups that are being statistically compared?	Yes	Materials and Methods, Figure legends

Sample definition and in-laboratory replication	Information included in the manuscript?	In which section is the information available? (Reagents and Tools Table, Materials and Methods, Figures, Data Availability Section)
In the figure legends: state number of times the experiment was **replicated** in laboratory.	Yes	Figure legends
In the figure legends: define whether data describe **technical or biological replicates**.	Yes	Materials and Methods, Figure legends

Ethics

Ethics	Information included in the manuscript?	In which section is the information available? (Reagents and Tools Table, Materials and Methods, Figures, Data Availability Section)
Studies involving **human participants**: State details of **authority granting ethics approval** (IRB or equivalent committee(s), provide reference number for approval.	Not Applicable	
Studies involving **human participants**: Include a statement confirming that **informed consent** was obtained from all subjects and that the experiments conformed to the principles set out in the WMA Declaration of Helsinki and the Department of Health and Human Services Belmont Report.	Not Applicable	
Studies involving **human participants:** For publication of **patient photos**, include a statement confirming that consent to publish was obtained.	Not Applicable	
Studies involving experimental **animals**: State details of **authority granting ethics approva**l (IRB or equivalent committee(s), provide reference number for approval. Include a statement of compliance with ethical regulations.	Not Applicable	
Studies involving **specimen and field samples:** State if relevant **permits** obtained, provide details of authority approving study; if none were required, explain why.	Not Applicable	

Dual Use Research of Concern (DURC)	Information included in the manuscript?	In which section is the information available? (Reagents and Tools Table, Materials and Methods, Figures, Data Availability Section)
Could your study fall under dual use research restrictions? Please check biosecurity documents and list of **select agents and toxins** (CDC): https://www.selectagents.gov/sat/list.htm	Not Applicable	
If you used a select agent, is the security level of the lab appropriate and reported in the manuscript?	Not Applicable	
If a study is subject to dual use research of concern regulations, is the name of the **authority granting approval and reference number** for the regulatory approval provided in the manuscript?	Not Applicable	

Reporting

The MDAR framework recommends adoption of discipline-specific guidelines, established and endorsed through community initiatives. Journals have their own policy about requiring specific guidelines and recommendations to complement MDAR.

Adherence to community standards	Information included in the manuscript?	In which section is the information available? (Reagents and Tools Table, Materials and Methods, Figures, Data Availability Section)
State if relevant guidelines or checklists (e.g., **ICMJE, MIBBI, ARRIVE, PRISMA**) have been followed or provided.	Not Applicable	
For **tumor marker prognostic studies**, we recommend that you follow the **REMARK** reporting guidelines (see link list at top right). See author guidelines, under 'Reporting Guidelines'. Please confirm you have followed these guidelines.	Not Applicable	
For **phase II and III randomized controlled trials**, please refer to the **CONSORT** flow diagram (see link list at top right) and submit the CONSORT checklist (see link list at top right) with your submission. See author guidelines, under 'Reporting Guidelines'. Please confirm you have submitted this list.	Not Applicable	

Data Availability

Data availability	Information included in the manuscript?	In which section is the information available? (Reagents and Tools Table, Materials and Methods, Figures, Data Availability Section)
Have **primary datasets** been deposited according to the journal's guidelines (see 'Data Deposition' section) and the respective accession numbers provided in the Data Availability Section?	Yes	Data Availability section
Were **human clinical and genomic datasets** deposited in a public access-controlled repository in accordance to ethical obligations to the patients and to the applicable consent agreement?	Not Applicable	
Are **computational models** that are central and integral to a study available without restrictions in a machine-readable form? Were the relevant accession numbers or links provided?	Not Applicable	
If publicly available data were reused, provide the respective **data citations in the reference list**.	Not Applicable	
